# Cancer-linked aggregation of p53 is driven by sequence-encoded frustration, solvation, and hydrophobic gating absent in its paralogs

**DOI:** 10.1038/s42004-026-02050-2

**Published:** 2026-05-16

**Authors:** Guilherme C. de Andrade, Ander F. Pereira, Paulo H. B. Ferreira, Karen D. da Cruz, Gileno S. de Sousa, Michelle F. Mota, Ronaldo J. de Oliveira, Leandro Martínez, Mayra A. Marques, Jerson L. Silva, Guilherme A. P. de Oliveira

**Affiliations:** 1 Institute of Medical Biochemistry Leopoldo de Meis, National Institute of Science and Technology for Structural Biology and Bioimaging, National Center of Nuclear Magnetic Resonance Jiri Jonas, Federal University of Rio de Janeiro, Rio de Janeiro, Brazil; 2 Laboratory of Structural Bioengineering Jerson Silva, Institute of Medical Biochemistry Leopoldo de Meis, Federal University of Rio de Janeiro (UFRJ), Rio de Janeiro, Brazil; 3 Institute of Chemistry and Center for Computing in Engineering & Science, Universidade Estadual de Campinas (UNICAMP), Campinas, Brazil; 4 Laboratório de Biofísica Teórica, Department of Physics, Institute of Exact, Natural Sciences and Education, Federal University of the Triângulo Mineiro, Uberaba, Brazil; 5 Faculty of Pharmacy, National Institute of Science and Technology for Structural Biology, Federal University of Rio de Janeiro (UFRJ), Rio de Janeiro, Brazil; 6 D’Or Institute for Research and Education (IDOR), Rio de Janeiro, Brazil

**Keywords:** Solution-state NMR, Proteins, Molecular modelling, Protein aggregation

## Abstract

Tumor suppressor p53 is uniquely prone to misfolding and aggregation, a property tightly linked to oncogenic mutations and absent in its paralogs, p63 and p73. Yet, the biophysical origins underlying divergent aggregation propensity remain incompletely understood. Here, we integrate fluorescence spectroscopy, high-pressure and chemical denaturation NMR, molecular dynamics simulations, and energetic frustration analysis to dissect the structural and dynamic discrepancies among p53 and its paralogs. Our results reveal that p53 harbors distinct surface-exposed solvation patches and cavity-prone regions promoting local conformational plasticity. Residue-level NMR analysis uncovers a broad, asymmetric distribution of signal intensity changes with pressure, correlating with discrete solvation patches and frustration-prone regions. These features coincide with highly frustrated contact networks and altered hydrophobic core gating, collectively facilitating aggregation-prone states. In contrast, p63 and p73 exhibit more uniform solvation, reduced frustration, and tighter core packing, conferring greater structural stability. These mechanistic differences directly link uneven hydration and compressibility to the heightened amyloid aggregation propensity of p53C. These contrasting energetic landscapes illustrate how evolutionary sequence divergence fine-tunes p53 for functional flexibility at the expense of stability, predisposing it to pathological aggregation. Our findings illuminate the structural determinants that distinguish pathological folding in p53 and may guide therapeutic stabilization strategies.

## Abstract (web summary)

Tumor suppressor p53 is prone to misfolding and aggregation linked to oncogenic mutations, which are absent in its paralogs p63 and p73. Here, the authors use advanced spectroscopy, NMR, and simulations to reveal distinct solvation patches and frustration-prone regions in p53, revealing the biophysical origins underlying divergent aggregation propensity.

## Introduction

The p53 family of tumor suppressors comprises p53, p63, and p73, which share structurally homologous DNA-binding domains (DBDs), also known as core domains, and hereafter referred to as p53C, p63C, and p73C, yet have evolved to fulfill distinct functional roles^1^. Ancestral functionalities of the p53 homolog Cep-1 in *Caenorhabditis elegans* include the regulation of meiotic chromosome segregation, highlighting its pivotal role in maintaining germline genome integrity^2^. Although Cep-1 mutations do not affect somatic apoptosis, they confer hypersensitivity to hypoxia and reduced survival under starvation, suggesting that p53-related functions were not restricted to the germline but also extended to somatic stress responses^2^. The emergence of vertebrates placed new evolutionary pressures on ancestral p53, as complex, proliferative somatic tissues demanded high-fidelity genome surveillance. In response to these selective forces, and first evidenced in cartilaginous fish^3^, a primitive p53-like gene underwent duplication, giving rise to specialized paralogs that assumed distinct but complementary roles: safeguarding genome integrity in both germline and somatic cells. While preventing unrestrained proliferation and tumorigenesis (p53 ancestral), it also orchestrates normal tissue and organ development during embryogenesis (a single p63/p73 ancestral).

Comparative analyses of the p53 DNA-binding domain (DBD) across bony vertebrates and humans have revealed extensive amino acid divergence, indicating that the *TP53* gene has evolved more rapidly than *TP63* and *TP73* to acquire specialized functions^3^. While p53 has emerged as the master regulator of the somatic genome, explaining the high prevalence of *TP53* mutations in malignant tissues, p63 and p73 have diverged to support distinct developmental programs, including morphogenesis, neurogenesis, and immune system maturation. For instance, p63 is abundantly expressed in the basal layers of epithelial tissues and plays a fundamental role in epithelial morphogenesis^4^. Mutations in the human *TP63* gene result in cleft palate, limb malformations, and skin disorders, collectively known as ectrodactyly-ectodermal dysplasia-clefting (EEC) syndrome; notably, these individuals exhibit a low incidence of cancer^4^. Mice lacking p73 exhibit hippocampal dysgenesis, hydrocephalus, chronic infections, inflammation, and impaired pheromone sensing, underscoring its essential role in neurodevelopment and immune regulation^5^. In contrast, these mice do not develop spontaneous tumors. Such striking divergence in physiological roles, despite conserved structural domains, provides a robust framework to investigate sequence-specific determinants underlying a pathological feature of p53-driven tumorigenesis: its increased propensity to aggregate upon mutation at hotspot sites^6–8^.

Notably, both our group and others have demonstrated that dissecting the determinants of p53 aggregation holds therapeutic potential in cancer^9,10^. In tumor cells, mutations at hotspot residues within the p53 core domain exacerbate structural destabilization^6^, ultimately leading to pathological aggregation^11–17^ and the gain-of-function transformation of p53 into an oncogenic protein^18–20^. While several small molecules are currently under investigation and have shown promise in mitigating mutant p53 aggregation^21–25^, we were the first to demonstrate the presence of aggregated mutant p53 in living glioblastoma cell lines^26^ and in breast cancer tissues^11^. More recently, the detection of elevated levels of p53 aggregates in the plasma of glioblastoma patients prior to surgery suggests the potential utility of these aggregates as early diagnostic biomarkers. Elucidating the sequence divergences among p53, p63, and p73, and their structural consequences for aggregation, may reveal critical surface-exposed platforms that could be targeted to prevent or detect early conversion to aggregated states^27^. An additional issue within the p53 family concerns the way these proteins interact when p53 acquires destabilizing cancer-associated mutations^28^. Evidence has shown that mutant p53 coaggregates with its paralogs p63 and p73, triggering a heat-shock response in cells^29^. More recently, mutant p53 has been shown to convert non-amyloid p63C and p73C condensates into amyloid-like structures, further supporting its role in initiating amyloid aggregation in its paralogs^30^.

**Fig. 1:**
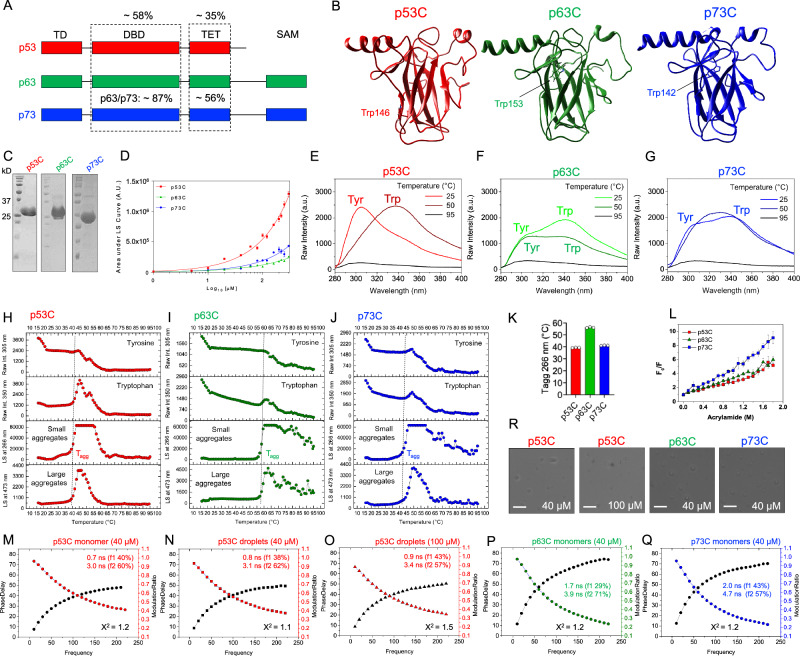
Aromatic environment and conformational heterogeneity in p53 family members. **A** Schematic representation of p53 family domains and sequence conservation. TD transactivation domain, DBD DNA-binding domain, TET tetramerization domain, SAM sterile alpha motif. **B** Ribbon representation of the p53 family DNA-binding core domains, highlighting the tryptophan location. **C** Representative SDS-PAGE showing ^15^N-enriched proteins. **D** Dot plot showing the area under the light scattering (LS) curve as a function of protein concentration. p53C displays a pronounced, exponential increase in scattering consistent with concentration-driven self-association, whereas p63C remains largely soluble, and p73C exhibits intermediate behavior. Fluorescence emission spectra for **E** p53C, **F** p63C, and **G** p73C acquired at 25, 50, and 95 °C, showing contributions from tyrosine (Tyr) and the single tryptophan (Trp). In the native state, fluorescence is dominated by tyrosine emission (~305 nm), while the single tryptophan is quenched. Upon thermal perturbation, loss of Tyr-to-Trp energy transfer leads to increased tryptophan emission (~350 nm), reporting local unfolding and solvent exposure of the Trp residue. **H**–**J** Stacked plots of tyrosine (305 nm) and tryptophan (350 nm) fluorescence emission, combined with static light scattering at 266 and 473 nm, plotted against increasing temperature. Early changes in Trp emission reflect local unfolding and solvent exposure, whereas the concomitant increase in light scattering indicates progression of aggregation. The simultaneous rise in Trp fluorescence and scattering demonstrates that unfolding and aggregation are coupled processes in these proteins. Dashed vertical lines indicate the aggregation temperature (Tagg). **K** Bar plot showing Tagg values. **L** Stern–Volmer plot from acrylamide quenching experiments for p53C, p63C, and p73C, probing solvent accessibility of the single tryptophan residue. Higher K_sv_ (Stern-Volmer constants) values indicate increased solvent exposure in the folded ensemble. **M**–**Q** Double-Y plots showing phase delay (lighter colors) and modulation ratio (darker colors) as a function of increasing harmonics. Chi-square (χ²) values correspond to two-exponential fittings. **R** Bright-field micrographs showing p53C, p63C, and p73C protein condensation into droplets. Data in (**D**, **K**, **L**) represent the mean ± s.e.m. of *n* = 3 independent experiments using the same protein batch. Scale bar, 20 μm.

All three p53 family members share an N-terminal transactivation domain, a central DNA-binding core domain, a tetramerization domain, and a C-terminal regulatory region (Fig. 1A). Unique to p63 and p73, however, is a C-terminal sterile alpha motif (SAM) domain, which contributes to protein stability and mediates specific protein–protein interactions^31^. The loss of this domain in p53 represents a major point of functional divergence from its paralogs, reinforcing the notion that domain architecture is a critical determinant of protein function. The tetramerization domain, common to all three proteins, consists of a dimer-of-dimers arrangement, forming a short β-strand and a primary α-helix (H1). While p63 and p73 share 56% sequence identity in this region, this similarity drops to ~35% when compared to p53 (Fig. 1A). A notable structural distinction lies in an additional α-helix (H2) that stabilizes the p73 tetramer, which is absent in p53^32,33^. The sequence identity of the central DNA-binding domains (DBDs) between p63 and p73 reaches 87%, whereas the comparison among all three proteins reveals only 58% identity (Fig. 1A). The underlying cause of how the sequence divergence in the p53 DBD, despite maintaining the same overall fold as its paralogs (Fig. 1B), results in increased conformational instability remains poorly understood. To uncover why p53, but not its paralogs, readily aggregates, we conducted a high-resolution residue-level analysis combining fluorescence spectroscopy, NMR, and molecular dynamics to identify the key sequence features and structural triggers that undermine p53 core stability and drive its pathological behavior.

Here, we demonstrate that distinct solvation patterns, local energetic frustration, and altered hydrophobic core gating collectively define the unique aggregation behavior of the p53 core domain in comparison to its p63 and p73 paralogs. Using an integrated approach, we reveal that specific surface patches and cavity-prone regions in p53C promote conformational plasticity while compromising stability. These features create a dynamic landscape that facilitates early structural transitions toward aggregation, offering mechanistic insight into how evolutionary divergence in p53 encodes its inherent instability linked to tumorigenesis.

## Methods

### Expression and purification

The gene encoding the DNA-binding domain (DBD) of human p53 was a gift from Cheryl Arrowsmith (p53C, addgene plasmid #24866), and p73 was a gift from Nicola Burgess-Brown (p73C, addgene plasmid #39077). The p63 sequence was synthesized using Genscript services (p63C, Genscript synthesis). We first optimized protein yields using four different *E.coli* strains and trace elements solution (100x) containing 5 g/L EDTA (Vetec #297), 0.83 g/L FeCl_3_ × 6 H_2_O (Vetec #V900278), 84 mg/L ZnCl_2_ (Vetec #573), 13 mg/L CuCl_2_ × 2 H_2_O (Vetec #V800129), 10 mg/L CoCl_2_ × 6 H_2_O (Vetec #900021), 10 mg/L H_3_BO_3_ (Vetec #263), 1.6 mg/L MnCl_2_ × 6 H_2_O (Vetec #567). Uniformly ^15^N-labeled proteins were expressed in *Escherichia coli* BL21-CodonPlus (DE3) cells. Cultures were initially grown in Luria–Bertani (LB, Kasvi #K251551) medium at 37 °C until an optical density at 600 nm of 1.0 was reached. Cells were harvested by centrifugation and resuspended in ^15^N-enriched M9 minimal medium supplemented with yeast nitrogen base without amino acids (Benton, Dickison and Company #233520). Protein expression was induced with 1 mM isopropyl β-D-1-thiogalactopyranoside (IPTG, Thermo Scientific #R0393) and carried out overnight at 25 °C under constant agitation.

Cells were harvested by centrifugation, resuspended in lysis buffer [50 mM Tris-HCl (Sigma #93352), 500 mM NaCl (Sigma #S7653), 5 mM dithiothreitol (DTT, Promega #V3155), 1 mM phenylmethylsulfonyl fluoride (PMSF, Sigma #P7626), pH 7.4], and lysed by sonication. Cell debris and insoluble material were removed by centrifugation. The soluble fraction containing p53C was purified using a HisTrap FF nickel affinity column (Cytiva #17525501) with elution performed through a linear imidazole gradient (Sigma #792527). The fusion tag was removed by thrombin (human source) digestion at 4 °C, followed by dilution and further purification on a Heparin Sepharose FF column (Cytiva #17099801).

The p63C was purified following a similar protocol, except that the final purification step was performed using a Superdex 75 size-exclusion column (Cytiva #28989333), yielding a highly homogeneous sample. The purification protocol for p73C has been previously described^34^. Briefly, the soluble fraction was loaded onto a HisTrap FF column, cleaved with TEV protease (recombinant production), and reapplied to the same column to remove the cleaved tag. The unbound fraction was collected as the purified protein.

We performed SDS-PAGE and size-exclusion chromatography to ensure the high purity of our preparations. For experiments, all final protein samples were dialyzed against a buffer containing 20 mM Tris-HCl (pH 7.4), 150 mM NaCl, 5 mM DTT, and 5 µM ZnCl₂ and concentrated using an Amicon Stirred Cell (Merck # UFSC05001) coupled with N_2_ and a 10,000 MWCO filtration disc (Merck #PLGC02510). To prevent protein aggregation, all concentrations never exceeded 350 μM. Proteins were freshly frozen at −80 °C in the presence of 5% glycerol (Sigma #G5516). Before each experiment, proteins were thawed on ice and then centrifuged at 20,000 × *g* and 4 °C for 15 min. Protein concentrations were determined spectrophotometrically at 280 nm using the following molar extinction coefficients: 17,420 M⁻¹ cm⁻¹ for p53C, 15,930 M⁻¹ cm⁻¹ for p63C, and 17,285 M⁻¹ cm⁻¹ for p73C.

### Light scattering measurements

Light scattering (LS) measurements were carried out using an ISS K2 spectrofluorometer (ISS Inc., USA). The p53C, p63C, and p73C proteins were prepared in 20 mM Tris-HCl (pH 7.4), 150 mM NaCl, 5 mM DTT, and 5 µM ZnCl₂, at concentrations ranging from 1 to 350 µM (1, 5, 10, 20, 40, 60, 80, 100, 120, 160, 200, 220, 250, 300, and 350 µM). Excitation was performed at 320 nm, and emission spectra were recorded from 300 to 330 nm with a slit width of 5 mm. Light scattering intensity was quantified as the area under the emission curve.

### Thermal experiments

Thermal unfolding experiments were conducted using the UNCLE multifunctional stability platform (Unchained Labs). Samples of p53C, p63C, and p73C were prepared in the same buffer at a final concentration of 15 µM. Thermal ramps were performed from 16 to 95 °C in 1 °C increments. At each temperature point, fluorescence emission spectra (300–420 nm) from tryptophan and tyrosine residues were acquired, along with static light scattering (SLS) at 266 and 473 nm.

### Quenching experiments

To probe the accessibility of aromatic residues, acrylamide (Sigma #A8887) quenching experiments were conducted on all three proteins. Protein samples were titrated with acrylamide concentrations ranging from 0 to 2 M, and intrinsic tryptophan fluorescence was measured using an ISS K2 spectrofluorometer (ISS Inc., USA). Fluorescence intensities were plotted as F₀/F versus quencher concentration *[Q]*, where F₀ is the fluorescence in the absence of acrylamide, and F corresponds to the fluorescence at each titration point. The Stern–Volmer constant (K_sv_) was obtained from the slope of a linear regression fit to the data as shown in Eq. 1:

1 $$\frac{{F}_{0}}{F}={K}_{{sv}}\left[Q\right]+1$$

### Frequency-domain lifetime measurements

Frequency-domain fluorescence lifetime measurements were performed using a ChronosDFD spectrofluorometer (ISS Inc.) equipped with a 280 nm LED excitation source and a WA335 long-pass emission filter. A total of 22 frequency harmonics were used. L-tryptophan (Sigma #T-0254) lifetimes were measured in the previously mentioned protein buffer using L-tyrosine (Sigma #T-3754) in water as a reference standard (3.27 ns). Protein lifetimes were determined relative to tryptophan (measured lifetime: 2.89 ns). Lifetime data were analyzed using Vinci 3.0 software (ISS Inc.) by fitting both phase delay and modulation ratio to a discrete exponential decay model. Quality of the fits was evaluated by analysis of residuals and chi-squared (χ²) values.

Frequency-domain lifetime measurements were performed with an instrumental phase and modulation accuracy corresponding to an effective temporal resolution is on the order of ~0.1 ns.

### Differential interference contrast microscopy (DIC)

DIC images were captured using an EVOS™ M5000 Imaging System microscope equipped with a 60× objective lens. p53C, p63C, and p73C were prepared in a buffer containing 20 mM Tris-HCl (pH 7.4), 150 mM NaCl, 5 mM DTT, and 5 μM ZnCl_2_. The proteins were then diluted with a 50% polyethylene glycol 4000 (PEG 4000 – Sigma #81240) stock solution to achieve a final PEG concentration of 15%. The final protein concentration was either 40 or 100 µM.

### NMR spectroscopy

The p53C, p63C, and p73C proteins were prepared at 300 µM in a buffer containing 20 mM Tris-HCl (pH 7.4), 150 mM NaCl, 5 mM DTT, 5 µM ZnCl₂, 10% D₂O, and 1 mM DSS (Cambridge Isotope Laboratories #DLM-32-1) as internal standard. The ^1^H–^15^N correlation peaks at ambient pressure (1 bar) and in the absence of denaturants were assigned by comparison with previously published data^35^ and/or entries in the Biological Magnetic Resonance Data Bank (BMRB accession numbers 11012 and 26551).

High-pressure NMR experiments were conducted on a Bruker Avance III 800 MHz spectrometer equipped with a 5 mm TXI probe at 298 K. Heteronuclear ^1^H–^15^N TROSY-HSQC spectra were acquired using zirconia NMR tubes and an Xtreme-60 syringe pump system (Daedalus Innovations) for pressure control. For p63C and p73C, pressure was incremented from 1 to 2000 bar in steps of 250, 400, 500, 600, 750, 900, 1000, 1250, 1500, 1750, and 2000 bar. For p53C, the pressure steps included: 1, 100, 175, 250, 400, 500, 600, 750, 900, 1000, 1250, 1500, 1750, and 2000 bar. Spectra were recorded after a 15-min pressure equilibration period at each step. Spectral acquisition parameters were as follows: p53C—1024 (F2 ^1^H) × 200 (F1 ^15^N) points, 32 scans; p63C—1024 × 256 points, 32 scans; p73C—1024 × 128 points, 12 scans.

Guanidine hydrochloride (Sigma #50950) titration experiments were performed on the same instrument and at the same temperature, using standard glass NMR tubes. ^1^H–^15^N TROSY-HSQC spectra were acquired at increasing concentrations of guanidine hydrochloride: 0, 0.1, 0.3, 0.5, 0.8, 1.0, and 2.0 M. Acquisition settings were: p53C—1024 × 200 points, 32 scans; p63C—1024 × 200 points, 24 scans; p73C—1024 × 200 points, 16 scans.

Chemical shift perturbations (CSPs, Eq. 2) and intensity changes were monitored across pressure and denaturant conditions, using the assigned TROSY-HSQC spectrum at 1 bar and 0 M guanidine as the reference state. Longitudinal (R₁) and transverse (R₂) relaxation rates were measured at 1 bar and 150 bar. R₁ experiments used relaxation delays of 0.1, 0.25, 0.5, 0.75, 1.0, and 1.2 s; R₂ delays were 17, 34, 51, 68, 85, and 102 ms. Experimental uncertainties were estimated based on the signal-to-noise ratios of the respective spectra. All spectra were processed using TopSpin 3.1 (Bruker) and analyzed with CCPNMR Analysis^36^. Overlapping cross-peaks were excluded from analysis.

2 $${CSP}={\left[{\left({\varDelta \delta }_{{\rm H}}\right)}^{2}+0.1{\left({\varDelta \delta }_{{\rm N}}\right)}^{2}\,\right]}^{1/2}$$

High-pressure NMR data were analyzed using residue-resolved ¹H chemical shift perturbations as a function of pressure. For each assigned residue in p53C, p63C, and p73C, chemical shifts were fitted using both linear and quadratic models. Fit quality was assessed by the coefficient of determination (R²), and nonlinear pressure responses were identified by calculating *Δ*R² = R²_quadratic_ − R²_linear_. Residues with R² ≥ 0.95 and *Δ*R² ≥ 0.05 were classified as exhibiting nonlinear pressure dependence, indicative of pressure-sensitive cavities or low-populated excited states. This analysis was applied consistently across all proteins to enable direct comparison of cavity-related responses within the p53 family.

### Molecular dynamic simulations

The simulated systems, comprising residues 91–289 of the p53 monomer and a Zn²⁺ ion in solution, were constructed using the Solution Builder module of the CHARMM-GUI web server^37^, based on the crystal structure of chain A from PDB ID 2XWR^38^. The p53 fragment was acetylated at the N-terminus and amidated at the C-terminus, then placed at the center of a cubic box of water with dimensions 7.3 nm × 7.3 nm × 7.3 nm. The system pH was set to 7.0, and NaCl ions were added to achieve charge neutrality and a physiological salt concentration of 0.15 M. The CHARMM36m force field was used, with a reference temperature of 293 K^39^.

Because pressure-dependent aggregation has been reported for p53C^38^, we performed microsecond-scale molecular dynamics (MD) simulations using the GROMACS 2021.2 package^40^ under both atmospheric pressure and high pressure (3000 bar). These simulations allowed us to investigate the structural determinants that initiate pressure-induced destabilization. Importantly, the simulation protocol used here was specifically to model systems under high-pressure conditions^41^. Energy minimization was carried out using the steepest descent algorithm until the maximum force in the system dropped below 1000.0 kJ mol⁻¹ nm⁻¹. Equilibration was performed in the NVT ensemble for 125 ps. Production runs were carried out in the NPT ensemble using the leap-frog integrator, with a 2 fs timestep, for a total duration of 1.5 µs. Coordinates were saved every 100 ps, and energy values were recorded every 2 ps.

All bonds involving hydrogen atoms were constrained using the LINCS algorithm. Van der Waals interactions were treated with a switching function that smoothly turned off the forces between 1.0 nm and 1.2 nm. Electrostatics were computed using the smooth Particle Mesh Ewald (PME) method, with a real-space cutoff of 1.2 nm. Temperature coupling was implemented using the velocity-rescaling thermostat with a time constant of 1.0 ps, applied to two separate groups: the protein–Zn²⁺ complex and the solvent (water and ions). Pressure coupling was applied isotropically using the stochastic cell rescaling algorithm, with a time constant of 5.0 ps.

To ensure the Zn²⁺ ion remained coordinated at its binding site (residues C176, H179, C238, and C242), a harmonic potential was applied to maintain its position during the simulations. The simulation protocol was based on previous studies conducted by our group^42–44^ (Table S2).

### Hydration analyses

The hydration structures of the p53 family members were obtained from previously described atomistic molecular dynamics simulations of p53, p63, and p73^44^, as well as from simulations conducted at high pressure, as described above. To characterize solvent distribution around each protein, we computed Minimum-Distance Distribution Functions (MDDFs) using the *ComplexMixtures.jl* package^45,46^ (v.2.10.1). MDDFs are particularly suited for describing solvation around irregularly shaped solutes such as proteins, as they account for molecular geometry by measuring the shortest distances between solvent and solute atoms. These functions provide a clear solvation-shell interpretation by mapping how solvent density varies as a function of the minimum distance from the solute surface. Formally, the MDDF is defined as the ratio between the observed solvent density at a given distance, *n*_*s*_*(r)*, and the expected density under an ideal-gas distribution, *n*_*s*_^*∗*^*(r)*:

3 $${g}_{{ps}}\left(r\right)=\frac{{n}_{s}\left(r\right)}{{n}_{s}^{* }\left(r\right)},$$

where *p* denotes the protein, *s* the solvent (water), and *r* the minimum distance between solute atoms and each solvent molecule. The ideal reference distribution *n*_*s*_^*∗*^*(r)* is generated by randomly placing solvent molecules in the simulation box while preserving the bulk solvent density. The bulk density of water was estimated from the region between 8 and 12 Å from the protein surface, where solvent properties are no longer significantly influenced by solute proximity^47^. To examine solvation at the residue level, we used the *ResidueContributions* function available in *ComplexMixtures.jl*, which decomposes MDDFs into individual per-residue contributions.

Residue-level hydration was quantified by integrating per-residue MDDF contributions up to 1.8 Å and 2.6 Å, corresponding to the first (hydrogen-bonding) and second (non-specific) solvation shells, respectively. To estimate statistical variability, two independent trajectories per protein were each divided into two temporal blocks, yielding four blocks per protein and allowing calculation of mean hydration scores and standard deviations for each residue. To enable normalized and objective comparison of solvation patterns across family members while avoiding cross-protein bias, hydration scores were internally standardized within each protein by conversion to Z-scores, defined as:

4 $${Z}_{i}=({H}_{i}-\langle H\rangle )/{\sigma }_{H},$$

where ${H}_{i}$ is the mean hydration score of residue *i*, and $\left\langle H\right\rangle$ and ${\sigma }_{H}$ are the mean and standard deviation of the hydration distribution of the same protein.

The statistical convergence of the coordination number of selected residues in p53, p63, and p73 was confirmed by block-averaging using MolSimToolkit.jl package (v.1.31.1).

### Frustration analysis

The frustration index quantifies how favorable a native residue-residue interaction is compared to a set of randomized decoy contacts at the same position, normalized by the variance of the energy distribution. In practice, this is achieved by systematically mutating the interacting residues to all possible amino acid combinations and recalculating the total energy of the system^48^. According to the principle of minimal frustration, proteins have evolved under evolutionary pressure to minimize energetic conflict and support a funneled folding landscape. Consequently, minimally frustrated contacts represent evolutionarily optimized interactions that strongly favor the native state over alternative configurations. However, local frustration is not necessarily deleterious; it can emerge through neutral drift or be conserved as a functional feature, especially in regions requiring flexibility or conformational change^48^. We performed frustration analysis of p53, p63, and p73 DNA-binding domains using PDB codes 2FEJ, 2RMN, and 2XWC, respectively, at the frustratometer server (http://frustratometer.qb.fcen.uba.ar/localizing_frustration).

Local energetic frustration was analyzed using contact-based frustration indices computed with the Frustratometer for each core domain, p53C, p63C, and p73C. To enable quantitative comparison while respecting the conceptual limitations of cross-protein frustration analysis, frustration indices were normalized internally within each protein by conversion to Z-scores. This intra-protein normalization preserves relative energetic differences and allows direct comparison of homologous residues across paralogs. Mean, median Z-scores, contact counts, and the fraction of highly frustrated contacts were used for statistical analyses.

### Reporting summary

Further information on research design is available in the Nature Portfolio Reporting Summary linked to this article.

## Results

### Distinct aromatic environment and conformational heterogeneity in p53 family members

We first optimized protein expression yields by testing various *E. coli* strains in the presence and absence of trace elements. Independent of trace element supplementation, the BL21-CodonPlus strain consistently produced higher yields compared to BL21(DE3), C43(DE3)pLysS, and Rosetta(DE3) (Fig. S1A). The enhanced performance of CodonPlus is likely due to its incorporation of additional tRNA genes recognizing rare arginine codons (AGA and AGG), which are notably abundant within the DNA-binding domain of *TP53*. Using this system, we achieved high-level expression of p53C, p63C, and p73C (Fig. 1C) to investigate differences in aggregation propensity by combining light scattering measurements with intrinsic aromatic residue fluorescence.

Given that p53C, p63C, and p73C each contain a single tryptophan and several tyrosine residues, we leveraged the sensitivity of tryptophan and tyrosine fluorescence to report on local structural perturbations. Aggregation behavior was monitored as a function of protein concentration (Fig. 1D) and temperature (Fig. 1E–K). Concentration dependence is particularly critical, as nuclear magnetic resonance experiments require protein concentrations in the high micromolar range. Upon increasing concentrations from 1 to 350 µM, p53C exhibited a marked, exponential increase in light scattering signal, consistent with concentration-dependent self-association (Figs. 1D and S1B–D). In contrast, p63C showed negligible aggregation under the same conditions, while p73C displayed intermediate behavior (Fig. 1D). These findings suggest that p53C harbors surface-exposed features that render its fold more prone to intermolecular interactions, promoting the formation of higher-order oligomeric species.

We next performed thermal ramping experiments using the UNCLE platform to monitor intrinsic fluorescence from aromatic residues and light scattering (Fig. S1E–G). Fluorescence profiles at 25 °C, 50 °C, and 95 °C revealed protein-specific features in the environment of their single tryptophan residues (Fig. 1E–G). In p63C and p73C, the tryptophans, Trp153 and Trp142, numbered based on PDBs 2RMN and 2XWC, respectively, are positioned at the beginning of the β-strand S2, whereas in p53C, Trp146 (PDB 2FEJ) is located at the end of strand S3 (Fig. S2A–C). In contrast, tyrosine residues are more spatially conserved across the three paralogs. Based on p53 numbering, conserved tyrosines include Tyr103 (N-terminal loop), Tyr126 (S2), Tyr163 (S4), Tyr205 (S6), Tyr220 (S7–S8 loop), and Tyr234 (S8). Tyr107 and Tyr236, however, are not conserved in p63C and p73C, where they are replaced by histidine and phenylalanine, respectively (Fig. S2F). Additionally, Phe134 in p53C is replaced by tyrosine in both p63C and p73C (Fig. S2F). The loop–sheet–helix (LSH) motif of these proteins consists of the L1 loop, the antiparallel β-strands S2 and S2′ located near the N-terminus, and the α-helix H2 at the C-terminal region (Fig. S2A–C). This structural module contains key residues that mediate direct DNA contacts, and thus, a certain degree of intrinsic flexibility is expected to facilitate optimal binding to DNA. Notably, unlike in p53C, the tryptophan residues in p63C and p73C are located within the LSH motif. These differences in the distribution of aromatic residues underlie the distinct fluorescence emission profiles observed (Figs. 1E–G and S1E–G).

At room temperature, p53C exhibited strong tyrosine fluorescence centered around 302–305 nm and a quenched tryptophan signal. In contrast, p63C and p73C displayed a broader emission peak at ~340 nm, most likely due to a mixed Tyr/Trp contribution, with a modest shoulder attributed to tyrosine fluorescence (Fig. 1F, G). Upon thermal ramping, we monitored emission at 305 nm (tyrosine) and 350 nm (tryptophan), along with light scattering at 266 and 473 nm, to track early and late aggregation events, respectively (Fig. 1H–J). Across all proteins, tyrosine emission declined slightly from 15 to 25 °C. However, a similar early decrease in tryptophan fluorescence was only observed in p63C and p73C, suggesting that the tryptophan in p53C resides in a more thermally shielded environment.

Interestingly, further increases in temperature resulted in synergistic increases in tryptophan fluorescence and light scattering at 266 and 473 nm, revealing that during temperature-induced unfolding, solvent exposure of the tryptophan is accompanied by protein aggregation (Fig. 1H–J). Therefore, the single tryptophan of the p53 family members acts as a sensitive reporter of early aggregation events. Due to the sharp bathochromic transitions observed for p63C and p73C, estimating thermal stability from emission spectra alone was less reliable. To address this, we adopted a 10% threshold change in the 266 nm scattering intensity to define the aggregation onset temperature (T_agg_) (Fig. 1K). T_agg_ values revealed that p53C begins aggregating at 39 °C, p73C at 41 °C, and p63C only at 55 °C, indicating greater intrinsic stability in p63C relative to its paralogs.

To assess the local solvent accessibility of the tryptophan residues in p53C, p63C, and p73C, we performed acrylamide quenching experiments (Fig. S1H–J) and analyzed the data using Stern–Volmer plots (Fig. 1L). The Stern–Volmer quenching constants (K_sv_), extracted from the linear fits, were 2.43 ± 0.39 M⁻¹ for p53C, 2.55 ± 0.25 M⁻¹ for p63C, and 4.23 ± 0.42 M⁻¹ for p73C. Since higher K_sv_ values indicate greater solvent exposure of the fluorophore, these results suggest that the tryptophan residue in p73C is significantly less shielded than in p63C and p53C, which displayed comparable values within the uncertainties. This observation is particularly striking given that both p63C and p73C contain the single tryptophan residue at equivalent structural positions, highlighting subtle yet meaningful differences in local side-chain environments between the two proteins.

To further probe the local environment and dynamic behavior of the tryptophan indole ring, we carried out frequency-domain fluorescence lifetime measurements for each protein (Figs. 1M–R and S1K, L). For this analysis, we considered measuring the tryptophan lifetimes of the monomeric and using previously defined crowding conditions in which the p53 family undergoes phase separation into droplets^49^. Analysis of phase delay and modulation ratio signals revealed that fitting with two discrete lifetimes yielded optimal residuals and chi-squared (χ²) values. Single-lifetime models resulted in poor fits, and adding a third lifetime did not significantly improve the goodness-of-fit and were therefore excluded. Tryptophan lifetimes for p53C were approximately 0.8 ns and 3.0 ns, while p63C exhibited lifetimes of about 1.7 ns and 3.9 ns. For p73C, the corresponding values were approximately 2.0 ns and 4.7 ns. As expected, both the lifetimes and the fractional contributions were different comparing p53C with p63C and p73C, as the tryptophan in p53C is facing a structurally distinct environment as those from p63C and p73C. In p53C, the two lifetimes contributed 40 and 60%, whereas p63C and p73C showed a skewed distribution, with ~29–43% in the shorter component and ~57–71% in the longer one. More strikingly, under conditions in which these proteins phase separate (15% polyethylene glycol), we noticed amenable lifetime changes in all three proteins, suggesting subtle conformational rearrangement of these proteins upon droplets formation (Fig. 1N, O, R). However, given the small magnitude of the lifetime changes, contributions from altered solvent viscosity and refractive index due to PEG addition cannot be excluded. These results suggest that the local environment surrounding Trp146 in p53C supports a broader conformational or rotational heterogeneity compared to the more rigid environments observed in p63C and p73C.

### Surface patches and hydrophobic gating reveal evolutionarily divergent sequence patterns in the p53 family

**Fig. 2:**
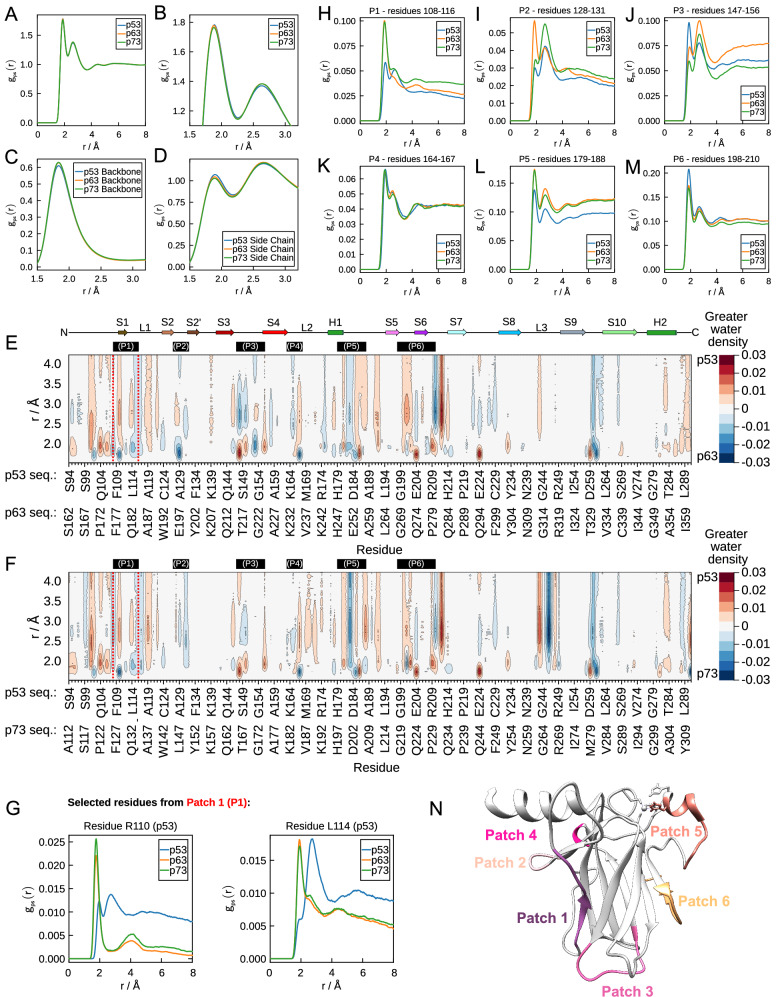
Altered solvation analysis in the p53 family. **A**–**D, G**–**M** Line plots showing minimum-distance distribution functions. Color maps showing the spatial density differences of water molecules across residues for **E** p53C-p63C and **F** p53C-p73C. Altered regions (patches P1–P6) are indicated by black rectangles. **N** Ribbon representation of the p53C structure, highlighting the identified solvation patches.

Previous molecular simulation studies have identified distinct segments of structural heterogeneity unique to p53, driven in part by an elevated incidence of backbone hydrogen bonds^44^. While the single tryptophan fluorescence effectively probes local environments, particularly within the loop–sheet–helix (LSH) motif, the method does not provide global insight into solvent interactions across the entire DNA-binding core. Here, we characterize the hydration structure of p53 (p53C), p63 (p63C), and p73 (p73C) to probe the differences in solvent accessibility among the paralogs and correlate these differences with the observed tryptophan fluorescence and NMR spectra. Previously published MD simulations were revisited to obtain the hydration structure around each residue, as characterized by the contributions to minimum-distance distribution functions^45^ (MDDFs, Figs. 2A–D, S3A, and S4).

Figure 2A shows the distribution of water molecules as a function of the distance from the protein surface. In all three cases, the profiles exhibit two main peaks: the first at ~1.8 Å, corresponding to hydrogen-bonding interactions, and the second at ~2.6 Å, reflecting non-specific water–protein interactions (the most prominent peaks are shown in Fig. 2B). Notably, the density in these hydration shells varies across the p53 family. The first solvation shell shows slightly higher water density for p53C compared to p63C and p73C, while the second shell displays higher density for p63C and p73C (Fig. 2B). Hydration quantitative analyses are visualized in Fig. S13.

The distributions were further decomposed into contributions from the protein backbone and side chains (Fig. 2C, D). Figure 2C shows that water interacts with the backbone primarily through hydrogen bonding, as evidenced by the peaks near 1.8 Å. Interestingly, the density of water molecules forming hydrogen bonds with the backbone is slightly higher for p63 and p73 than for p53. In contrast, side-chain interactions in p53 contribute more prominently to hydrogen bonding compared to p63 and p73 (Fig. 2D). The non-specific interactions between water and side chains, which dominate the second solvation shell, appear similar across all three proteins and primarily account for the second peak of the total MDDF (Fig. 2A, B). Water interactions with the backbone of proteins tend to be destabilizing, consistently with p53 displaying a folded basin with greater structural heterogeneity.

Figure 2E, F provides a detailed molecular view of the variations in water density around each residue of p53 and its paralogs based on per-residue contributions from the MDDFs. In these maps, red indicates higher water density in p53, while blue highlights regions where p63 (Fig. 2E) or p73 (Fig. 2F) show greater solvent accumulation. This analysis revealed six distinct surface patches (P1–P6) within the first two-thirds of the core domain that most strongly influence solvation in p53 compared to its paralogs. Notably, these regions correspond to segments that are poorly conserved across the family, suggesting that local protein–water interactions may have served as a selective pressure shaping the modern p53 sequence.

Patch 1 (P1; residues 108–116: GFRLGFLHS) spans the S1 strand and adjacent loops. In p53, R110 and L114 predominantly engage in non-specific interactions, whereas the corresponding residues in p63 (D178 and Q182) and p73 (E128 and Q132) participate in hydrogen bonding (Fig. 2E, F). This pattern is consistent with 1D per-residue contributions to the total MDDF in p53, p63, and p73 (Fig. 2G). Note that, at short distances (~1.8 Å), peaks for p63 and p73 are notably greater than for p53, while the opposite trend is observed at longer distances. Patch 2 (P2; residues 128–131: PALN), located in the S2–S2′ loop, exhibits strong hydrogen bonding in p63 (E197) but only dispersive contacts in p53 (A119) and p73 (L147) (Fig. 2E, F). Patch 3 (P3; residues 147–156: VDSTPPPGTR), within the S3–S4 loop, features D148 in p53 as a prominent hydrogen-bond acceptor, while the equivalent region in p63 (M216) favors weaker interactions (Fig. 2F, G). The most remarkable solvation feature of Patch 4 (P4; residues 164–167: KQSQ), located in loop L2, is that Q167 in p53 primarily engages in non-specific interactions, whereas the corresponding residues in p63 (E235) and p73 (E185) predominantly form hydrogen bonds (Fig. 2E, F). Patch 5 (P5; residues 179–188: HERCSDSDGL), which includes helix H1 and its downstream loop, shows increased water density at distances consistent with hydrogen bonding in p53, particularly around the charged residues R181 and D186. In contrast, the corresponding positions in p63 (L249 and G256) and p73 (L199 and G206) are occupied by non-polar residues. Consistently, water solvates arginine (in both the first and second solvation shells) and glutamate (in the first shell) more favorably in p63 (R251) and p73 (R201) than the corresponding serines (S183 and S185) in p53. Patch 6 (P6; residues 198–210: EGNLRVEYLDDRN), spanning the S5–S6 loop and strand S6, is characterized by stronger non-specific interactions (e.g., L201) and hydrogen bonding’s (E204 and R209) in the p53 relative to p63 (S271, Q274, and P279) and p73 (N221, Q224, and P229) (Fig. 2E, F). In contrast, hydrogen bonding is more prominent at position 201 in both p63 (S271) and p73 (Q274), while in positions 204 and 210 (Q274 and I280 in p63; Q224 and V230 in p73), residues primarily engage in non-specific interactions.

Figure 2H–M shows the contribution of residues from patches P1 to P6 in p53, p63, and p73 to the water MDDF. Although the density maps clearly reveal distinct solvation features across these regions in the p53 family, the overall contributions of residues from P4 and P6 vary only slightly among the three proteins (Fig. 2K, M). In contrast, water tends to solvate P1, P2, and P3 more efficiently in p63 and p73 than in p53 (Fig. 2H–J). A noticeable exception is observed in the first peak in Fig. 2J, which is higher for p53. This is likely due to the presence of more hydrophilic residues at positions 148, 150, and 156 (D148, T150, and R156), compared to the corresponding residues in p63 (M216, P218, and V224) and p73 (S166, 168, and A174).

To investigate the relationship between solvation anomalies and energetic frustration within the p53 family, we employed the Frustratometer suite to identify highly frustrated contacts (Fig. S3B–E). Frustration analysis across the p53 family revealed that while patches 1, 2, and 6 contain only a few highly frustrated residues, patch 3 is densely enriched in frustrated contacts within p53C (Fig. S3B–E). In contrast, the corresponding region in p63C and p73C, located in the S3–S4 loop, exhibits substantially lower frustration levels (Fig. S3D, E). Interestingly, canonical DNA-binding motifs such as the LSH motif and loop L3 are predominantly minimally frustrated in p53C, diverging from prior reports suggesting that catalytic and ligand-binding interfaces often exhibit elevated frustration^48^. Conversely, the DNA-binding surfaces of p63C and p73C display scattered frustrated residues, suggesting distinct energetic architectures across the paralogs (Fig. S3D, E). Taken together, the co-occurrence of altered solvation, elevated frustration, and low sequence conservation at patch 3 underscores this region as a dynamic and potentially maladaptive feature unique to p53C, distinguishing it from its paralogs and possibly contributing to its enhanced aggregation propensity and functional fragility. Statistical summaries of the normalized frustration distributions are reported in Fig. S14.

To further investigate the sequence-based determinants underlying the increased aggregation propensity of p53, particularly exacerbated in cancer-associated mutants, we analyzed the residues contributing to the hydrophobic shielding of the β-sandwich core (Fig. S2D, E). The DNA-binding domains of the p53 family adopt a compact β-sandwich architecture, consisting of two antiparallel β-sheets that enclose a dry hydrophobic core. Sheet 1 comprises strands S1, S3, S5, and S8, while sheet 2 is formed by S4, S6, S7, S9, and S10. Most of the core-facing residues in these strands are strongly hydrophobic and critical for structural stability. In contrast, S1, S2, S2′, and S6 contribute less to the hydrophobic core in all family members.

Notably, we identified a family-specific gating mechanism at both the upper and lower ends of the β-barrel, in which variations in hydrophobic residues may modulate the shielding efficiency of the core and influence conformational lability. At the bottom gate, the L145–V157 pair in p53C lies 3.6 Å apart and forms a partially shielded interface (Fig. S2E, bottom). In p63C, L145 is replaced by isoleucine, which extends the hydrophobic interaction and improves sealing at this region. Beyond the L145–V157 contact, an additional hydrophobic interaction occurs between strands S7 and S8, involving residues V218 and I232 in p53C and p73C (Fig. S2E, bottom). In p63C, both positions are occupied by valines, maintaining the hydrophobic sealing of this interface (Fig. S2E). While the physicochemical difference between isoleucine and valine is modest, such variation may subtly affect side-chain packing and shielding efficiency at the base of the core.

At the top gate, the Y236–V274 contact in p53C lies 3.3 Å apart and contributes to core capping (Fig. S2E, top). However, in both p63C and p73C, Y236 is replaced by phenylalanine, increasing local hydrophobicity and shielding (Fig. S2E). Moreover, residue T253 in p53C, a polar threonine facing the core and 3.7 Å from I295, is replaced by a bulkier, non-polar isoleucine in p63C and p73C (Fig. S2E), contributing additional hydrophobic coverage at the core entrance. Together, these findings reveal a multilayered mechanism by which p53 diverges from its paralogs: altered solvation patterns in non-conserved surface patches, elevated local frustration in dynamic regions, and reduced hydrophobic gating at the β-sandwich boundaries collectively define a structurally fragile and aggregation-prone landscape.

### NMR- and MD-based mapping of local hydration under pressure

**Fig. 3:**
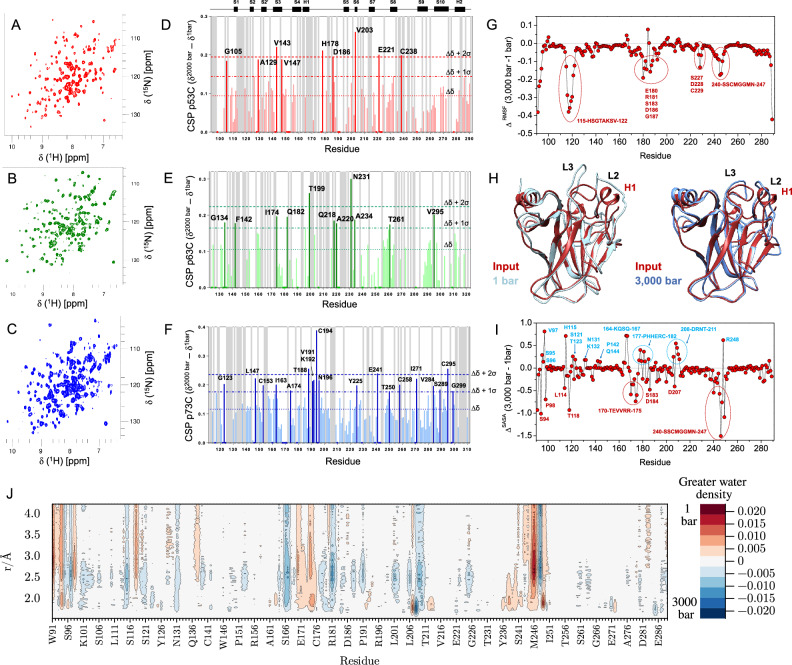
Chemical shift perturbation analysis and MD simulations in the p53 family. ^1^H–^15^N TROSY-HSQC spectra of **A** p53C, **B** p63C, and **C** p73C. **D**–**F** Bar plots showing chemical shift perturbation (CSP) values for 2000 bar vs. 1 bar, plotted as a function of residue number for p53C (red), p63C (green), and p73C (blue). Dotted, dash-dotted, and dashed horizontal lines represent the average (Δδ), average + 1 standard deviation (Δδ + 1σ), and average + 2 standard deviations (Δδ + 2σ), respectively. Ambiguous or unassigned H–N correlations are shown in gray; proline residues are depicted as circles. Secondary structure elements are indicated by black rectangles at the top. Residues exceeding Δδ + 1σ and Δδ + 2σ thresholds are highlighted. **G** Root mean square fluctuation differences (ΔRMSF) per residue of p53C comparing simulations at 3000 bar versus 1 bar. Negative values indicate reduced flexibility under high pressure. **H** Ribbon representation of the p53C input structure overlaid with the final MD frames at 1 bar and 3000 bar, highlighting pressure-induced structural rearrangements. L2, L3, and H1 stand for loop 2, loop 3, and helix 1. **I** Differences in solvent-accessible surface area (ΔSASA) per residue of p53C between simulations at 3000 bar and 1 bar. Positive values indicate increased solvent exposure at high pressure. **J** Spatial density maps of water molecules surrounding p53C residues, illustrating changes in hydration patterns between 1 bar and 3000 bar simulations. Blue regions indicate increased hydration at 3000 bar, while red regions indicate increased hydration at 1 bar.

**Fig. 4:**
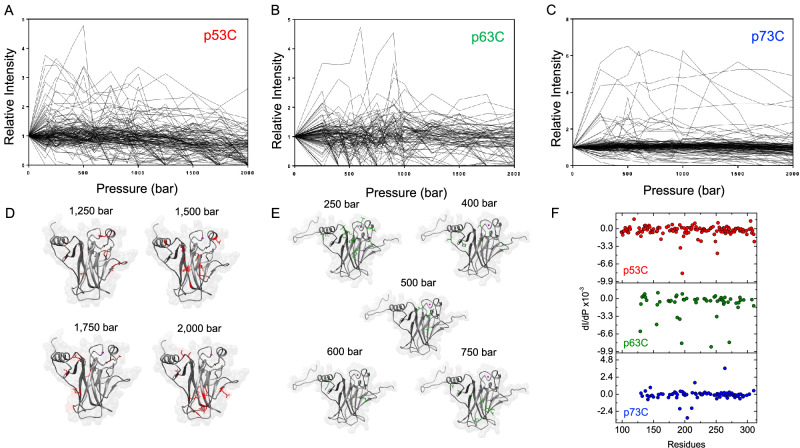
Pressure-dependent intensity profiles in the p53 family. Line plots showing the relative signal intensities of each assigned residue, normalized to the spectrum acquired at 1 bar, for **A** p53C, **B** p63C, and **C** p73C. Ribbon and surface representations of **D** p53C and **E** p63C, highlighting in red the residues whose signals disappeared at the corresponding pressure points. **F** Stacked plots showing the dI/dP values for p53C, p63C, and p73C.

Pressure is a robust tool for probing local unfolding, water infiltration, and the formation of intermediate states, some of which are associated with pre-amyloidogenic transitions and pathological protein aggregation^50,51^. To investigate structural dynamics and early unfolding events in the p53 family core domains, we monitored chemical shift perturbations (CSPs) (Figs. 3A–F, S5A–F and S9A–L) and residue-specific signal intensities (Fig. 4) in ^1^H–^15^N TROSY-HSQC spectra (Fig. 3A–C) upon titration with hydrostatic pressure (Fig. 3D–F) and guanidine hydrochloride (Fig. S10). Initial assignments were confirmed using published data and BMRB entries (accessions 11012 and 26551) (Figs. S6–S8). A total of 101 out of 201 (p53C, Fig. S6), 112 out of 200 (p63C, Fig. S7), and 123 out of 201 (p73C, Fig. S8) residues were tracked under pressure; and 126, 138, and 137 residues, respectively, during GdnHCl titrations. To obtain reliable high-pressure NMR spectra, we optimized our p53C sample preparations to eliminate concentration-induced aggregation prior to titration (see “Methods”).

CSPs were quantified as the differences between spectra at 2000 vs. 1 bar (Fig. 3D–F) and 0.8 vs. 0 M GdnHCl (Fig. S10A–C). Statistically significant perturbations (Δδ ≥ mean + 1σ or 2σ) were mapped onto each protein (Fig. S9A–L). Under high pressure, p53C showed localized perturbations at residues such as G105, A129, V143, D186 among others (Fig. 3D); p63C had broader shifts including F142, Q182, T199, N231, V295 among others (Fig. 3E); and p73C displayed the most extensive CSPs (Fig. 3F). Guanidine-induced shifts followed distinct patterns: p53C was affected in residues near the β-sandwich core (e.g., R110, C124, R174) (Fig. S10A), while p63C showed distributed CSPs with notable shifts at T130, V140, A184, S211, C273, C308 among others (Fig. S10B). Interestingly, p73C exhibited minimal CSPs, with only three residues (A140, N259, D305) significantly perturbed (Fig. S10C).

Given the sensitivity of p53C to high-pressure conditions reported in prior studies^38^, we compared simulations at 1 bar and 3000 bar to identify its structural features of destabilization (Fig. S11A–D). Flexibility per residue was evaluated by calculating the average Cα positional fluctuations (root mean square fluctuation, RMSF) over time. Comparison of ΔRMSF (3000–1 bar) revealed a global reduction in mobility, consistent with pressure-induced compaction (Fig. 3G). Notably, negative ΔRMSF outliers clustered at residues 115–122 (C-terminal to patch 1), within patch 5 (E180, R181, S183, D186, G187), and along loops L3 (240–247) and S227–C229, indicating enhanced local compressibility. Structural snapshots comparing initial and final MD frames at both pressures showed pronounced deformation of loops L2 and L3 and unfolding of helix H1 in patch 5 (Fig. 3H).

To assess how pressure affects hydration, we calculated per-residue changes in solvent-accessible surface area (ΔSASA) and MDDF. Positive values of ΔSASA indicate increased solvent exposure at 3000 bar (Fig. 3I), while in the MDDF Fig. 3J, increased hydration at 3000 bar corresponds to the blue regions. Strikingly, residues showing increased ΔSASA predominantly mapped to non-conserved family-specific patches. These include H115, S121, and T123 (patch 1); N131 and L132 (patch 2); full hydration of patch 4 (164–167); and residues 177–182 and 208–211 in patches 5 and 6, respectively. Conversely, a few segments, such as 170–175 and part of loop L3 (240–247), exhibited decreased SASA under pressure. These changes in protein–water interactions were also observed in the per-residue MDDF. These results suggest that specific loop segments in p53C become increasingly hydrated under high pressure, and that altered solvation patterns within poorly conserved regions may have played a key role in shaping p53’s evolutionary trajectory and aggregation propensity.

Changes in peak intensity reflected residue-specific destabilization. Relative intensities were calculated as the ratio of each residue’s signal under denaturing versus native conditions (Figs. 4A–F and S10D–F). In p53C, signal loss was prominent at higher pressures (1250–2000 bar), suggesting pressure-induced local unfolding or aggregation (Fig. 4A, B). Upon depressurization, the p53C NMR tube showed a cloudy solution, indicative of strong aggregation. In contrast, p63C exhibited signal loss at lower pressures (250–750 bar), indicating an earlier unfolding onset, but its spectra were fully reversible after pressure release (Fig. 4C, D). p73C remained largely unaffected throughout, confirming its higher resistance to pressure-induced perturbation (Fig. 4E, F). Mapped signal losses revealed distinct unfolding profiles: p53C displayed diffuse signal attenuation throughout the domain, consistent with non-cooperative structural destabilization (Fig. 4B). p63C showed a more ordered unfolding: initial changes localized near the Zn²⁺-binding region (250–400 bar), followed by the central β-sheet (500 bar), and distal sites (600–750 bar) (Fig. 4D). Only three residues in p73C (K183, A211, G246) showed any pressure-sensitive signal loss.

To further quantify residue-level pressure sensitivity, we calculated the first derivative of intensity with respect to pressure (dI/dP) across the pressure range (Fig. 4F). This parameter captures the changes in NMR signal intensity as pressure increases, providing a quantitative fingerprint of local volume compressibility and conformational lability. Notably, p53C exhibited a broad and asymmetric distribution of dI/dP values, with pronounced signal changes concentrated at residues located in solvation patches P3 and P5 (e.g., D148, R156, D186), regions previously identified as both highly frustrated and solvent-exposed. In contrast, p63C and p73C displayed narrower and more symmetric dI/dP profiles, consistent with their more cooperative unfolding and structural resilience. The heterogeneous dI/dP response in p53C aligns with its known tendency to form multiple aggregation-prone intermediates and supports the notion that local compressibility contributes to the early destabilization events that facilitate amyloid aggregation.

Under guanidine titration, p63C again proved more resistant, maintaining high signal intensities across most residues (Fig. S10E). In contrast, p73C showed progressive signal loss even at lower concentrations (0.3–0.8 M), highlighting a differential susceptibility to chemical versus mechanical denaturation (Fig. S10F). Residues that lost signal in p53C (Fig. S10D) were mapped onto its 3D structure at increasing guanidine concentrations (Fig. S10G), revealing clustering of unfolding-prone regions around the hydrophobic core, Zn²⁺-binding site, and solvent-exposed loops. A concise comparison of hydrostatic pressure versus chemical denaturation effects across the p53 family is summarized in Table S1.

**Fig. 5:**
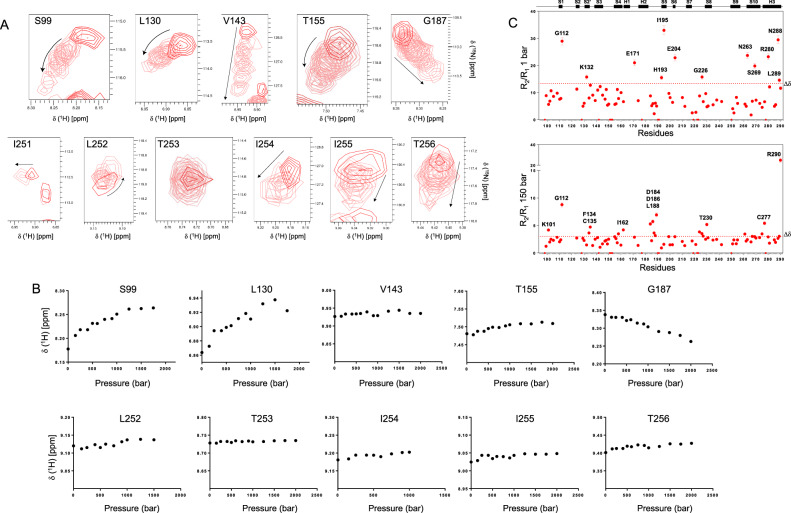
Dynamic properties in p53C. **A** Representative ^1^H–^15^N correlations displaying distinct pressure-dependent behaviors: nonlinear (S99, L130, T155); predominantly linear ^1^H–^15^N shifts (G187); linear ^15^N shifts (V143); highly pressure-sensitive (I251); highly pressure-insensitive (L252, T253); and modest linear shifts (I254, I255, T256). **B** Dot plots showing ^1^H chemical shift changes as a function of pressure. **C** Dot plots of the R₂/R₁ relaxation ratio plotted against p53C residue number at 1 bar (top) and 150 bar (bottom). Values exceeding the average (Δδ) are highlighted. Proline residues are indicated as small circles.

To gain further insight into local conformational plasticity and its potential implications for aggregation in p53C, we analyzed the pressure-dependent ^1^H chemical shift behavior of selected residues in p53C (Fig. 5A, B), focusing on nonlinear responses that may indicate the presence of sparsely populated excited states. Akasaka and colleagues previously demonstrated that such nonlinear chemical shift perturbations under increasing hydrostatic pressure can serve as markers of low-occupancy conformers, particularly in regions near internal cavities^52^. In our analysis, serine 99 (S99) and leucine 130 (L130) exhibited pronounced nonlinear ^1^H chemical shift responses, whereas valine 143 (V143), threonine 155 (T155), and glycine 187 (G187) showed more modest but still detectable pressure-sensitive behavior. Interestingly, residues within the amyloidogenic ILTIITL segment (residues 251–257) displayed a more linear response pattern, suggesting a relatively shielded and conformationally restricted environment; however, I251 stood out by exhibiting marked line broadening even under mild pressure (150 bar), indicating local heterogeneity or exchange dynamics (Fig. 5A). Although nonlinear pressure responses were detected in all three proteins, the nonlinear residues identified in p53C preferentially localize within or in close spatial proximity to the solvation patches (P1–P6), whereas in p63C and p73C they lack systematic association with these hydration-defined regions (Fig. S15). Unfortunately, direct comparison of homologous residues was not always feasible, as data for specific homologous positions were missing in one or more paralogs due to early signal loss and/or the lack of unambiguous resonance assignment.

Backbone ^15^N relaxation measurements performed at 1 bar and 150 bar revealed pronounced global changes in p53C dynamics under hydrostatic pressure (Figs. 5C and S12). Most notably, the average transverse relaxation rate (R₂) decreased markedly from 13.33 ± 7.31 s⁻¹ at 1 bar to 4.57 ± 3.8 s⁻¹ at 150 bar. In contrast, the longitudinal relaxation rate (R₁) remained essentially unchanged, with average values of 1.65 ± 0.98 s⁻¹ at 1 bar and 1.63 ± 0.46 s⁻¹ at 150 bar. As a result, the R₂/R₁ ratio showed a generalized reduction across the protein, consistent with an increase in structural flexibility on the picosecond-to-nanosecond (ps–ns) timescale. This dynamic signature suggests that exposure to 150 bar induces a relaxation of tertiary contacts in p53C, potentially indicative of a multisite global unfolding event. This behavior stands in contrast to what has been observed for other p53 family members under pressure: p63C primarily exhibits localized unfolding transitions, while p73C displays enhanced resistance to pressure-induced perturbation, maintaining a compact and stable structure even at elevated pressures. The global decrease in the R₂/R₁ ratio in p53C thus reflects a distinct destabilization mechanism likely related to its lower hydrophobic core integrity and higher intrinsic energetic frustration at key patches. The R₂/R₁ ratio also offers insights into conformational exchange processes occurring on slower microsecond-to-millisecond timescales. At 1 bar, a subset of residues, including G112, K132, E171, H193, I195, E204, G226, N263, S269, R280, N288, and L289, displayed elevated R₂/R₁ values relative to the average, suggesting involvement in exchange dynamics. Under 150 bar, a different set of residues became dominant in this regime, including K101, G112, F134, C135, I162, D184, D185, L188, T230, C277, and R290. This redistribution of conformational exchange-prone residues suggests a pressure-induced reorganization of the conformational landscape, with new flexible or partially disordered regions emerging under mechanical perturbation.

## Discussion

The relationship between water and the p53 protein family appears to be an evolutionary adaptation intimately tied to their functional requirements. As transcription factors, these proteins demand a high degree of flexibility and dynamic behavior, enabling promiscuous interactions with diverse DNA response elements while preserving specialized regulatory activity. A hallmark of such functional versatility is the presence of intrinsically disordered regions, where the lack of stable secondary structure permits broad interaction potential and efficient signal propagation. The p53 family embodies both hallmarks: disordered N- and C-terminal segments that govern transactivation and post-translational regulation, and a central DNA-binding domain characterized by multiple flexible loops, minimal secondary structure, and structurally decoupled functional units, such as the loop–sheet–helix (LSH) motif, the β-sandwich scaffold, and the Zn²⁺-coordination sphere. These structural features are conserved across the p53 family; yet, their primary sequences have diverged substantially, allowing each paralog to fulfill distinct functional roles. The sequence divergence indicates that selective forces have shaped their evolutionary paths.

In this study, we extended our understanding of how protein–water interactions and hydrophobic gating influence structural behavior within the p53 family and contribute to their functional specialization. We identified solvent-exposed surface patches, predominantly located within flexible loop regions and poorly conserved among family members, that display distinct solvation profiles in p53C compared to p63C and p73C (Fig. 2). These regions, while structurally tolerated across the family, exhibit markedly altered water interactions in p53C. Even more strikingly, several pressure-sensitive residues identified by NMR and MD simulations at 3000 bar are located near or within these patches in all three proteins (Fig. 3), reinforcing the notion that solvation may have acted as an evolutionary driver of sequence divergence. Additionally, highly frustrated residues in p53C are densely clustered within solvation patch 3, highlighting an evolutionary trade-off: the tolerance of maladaptive segments, decreased stability, and imminent aggregation propensity in exchange for enhanced specificity and conformational flexibility required for multilayered molecular recognition.

Pressure, when coupled with fluorescence and NMR spectroscopy, is a powerful tool in physical chemistry for probing local unfolding, water infiltration, and the formation of intermediate states, some of which are associated with pre-amyloidogenic transitions and pathological protein aggregation^50,51^. Seminal high-pressure studies demonstrated that compression modulates protein–solvent hydrogen bonding, facilitating water penetration and formation of “wet globule” intermediates^53^. We previously showed that p53C exhibits pressure-induced aggregation above 3000 bar and at concentrations as low as 5 µM, while p63C and p73C remained aggregation-resistant under similar conditions^44^. Notably, even a highly stabilized p53C quadruple mutant (QM: M133L, V203A, N239Y, N268D), which substitutes Met133 and Val203 with the equivalent residues found in p63C/p73C^54^, failed to prevent aggregation^44^, indicating that increased thermodynamic stability alone does not mitigate p53C’s aggregation propensity. Pressure-induced aggregation was only abrogated when sub-denaturing concentrations of guanidine hydrochloride (0.5–0.8 M) were applied^55^. Furthermore, a rationally designed hexamutant (HM)^56^, incorporating the QM plus two additional substitutions (Y236F and T253I) that introduce hydrophobic core residues from p63C/p73C into p53C hydrophobic gating residues, showed a drier internal core and reduced aggregation^57^. When compared across p53C, HM p53C, p63C, and the destabilized cancer mutant p53C-Y220C, which harbors a surface cleft prone to hydration, only p53C and p53C-Y220C demonstrated significant water infiltration^57^. This concept aligns with classical folding theories. Garcia and Onuchic provided compelling evidence that desolvation is an active step in protein folding^58^. They identified a near-native intermediate of an SH3 domain with a solvated hydrophobic core that required a final desolvation step, expelling water from the interior to reach a fully folded, compact state^59^. These findings support the view that p53C’s intrinsic susceptibility to water infiltration is rooted in its non-conserved loop regions and altered hydrogen bonding network, features that appear to facilitate water penetration into otherwise hydrophobic regions, ultimately promoting aggregation through solvent-mediated destabilization, even in the presence of enhanced core stability.

Given that NMR spectroscopy requires relatively high protein concentrations, we first examined whether p53C aggregation is concentration-dependent, as this could compromise pressure-induced unfolding experiments. Indeed, in contrast to p63C and p73C, p53C exhibited a pronounced increase in light scattering with rising concentration (Fig. 1D), consistent with a higher propensity for self-association. Remarkably, several residues located within or adjacent to solvent-sensitive patches (P1–P6) exhibited significant chemical shift perturbations under pressure across all three proteins, suggesting active participation of these regions in water-mediated structural transitions. Notably, Gly105 in p53C and its counterparts Gly134 and Gly123 in p63C and p73C (near Patch 1); Ala129 in p53C and Leu147 in p73C (Patch 2); Val147 in p53C, Ala184 in p63C, and Ala174 in p73C (Patch 3); His178 and Asp186 in p53C, Gln218 and Ala220 in p63C, and Asn196 in p73C (Patch 5); and Val203 in p53C, along with Asn231 and Ala234 in p63C and Tyr225 in p73C (Patch 6), all displayed pressure-sensitive NMR shifts. These observations point to a recurrent role for these surface-exposed segments in mediating protein–water interactions. The fact that these residues are structurally analogous yet sequence-divergent among the three paralogs suggests that differential solvation across evolution may have shaped their primary structures to either favor or resist aggregation-prone states.

Fluorescence emission measurements targeting tyrosine residues and the single tryptophan in each protein provided valuable local insights into the loop–sheet–helix (LSH) motif architecture. This motif is composed of the L1 loop, the antiparallel β-strands S2 and S2′, and the α-helix H2. In p63C and p73C, the single tryptophan resides within strand S2, whereas in p53C it is positioned at the end of strand S3 (Fig. S2A–C). Helix H2 contains two critical DNA-contacting residues, R280 and R283, while loop L1 establishes DNA interactions through K120^60^. Additional stabilizing elements for DNA binding are in the S10–H2 connector and the L3 loop^60^. Collectively, these regions enable precise adjustments for DNA engagement. Since these contacts are essential for maintaining DNA-binding affinity, the degree of structural flexibility or rigidity within the LSH motif likely reflects distinct mechanisms by which each p53 family member recognizes and responds to specific DNA elements.

**Fig. 6:**
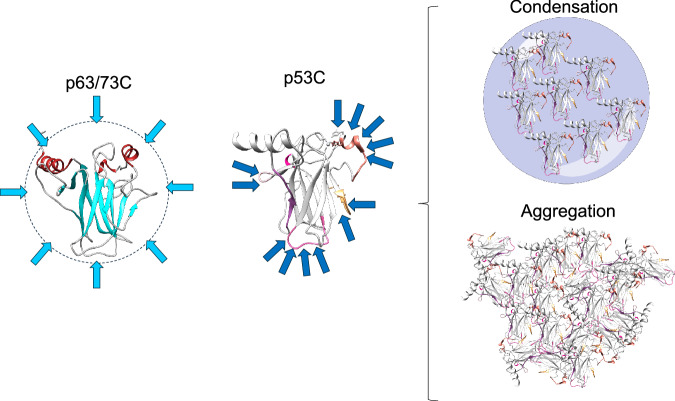
Schematic representation of the key findings. In contrast to p63C and p73C, p53C displays an uneven solvation landscape concentrated in non-conserved surface patches (colored), which enhances its susceptibility to phase separation and aggregation through solvent-mediated destabilization.

Frequency-domain fluorescence lifetime measurements revealed different lifetime components across the p53 family, reflecting variations in the local environments of the tryptophan residues. The use of elevated p53C concentrations and polyethylene glycol to simulate a crowded cellular environment promoted its condensation into liquid-like droplets. Despite this phase transition, only subtle conformational rearrangements were detected in the vicinity of the single tryptophan residue, as indicated by modest changes in fluorescence lifetimes. The short-lifetime p53C component shifted from 0.7 ns in the monomeric state to 0.8 ns within condensates and further increased to 1.0 ns upon doubling the protein concentration. These findings suggest that condensation induces a modest compaction of the p53C structure, resulting in a more rigid and less solvent-accessible environment around the tryptophan (Fig. 6). To our knowledge, this is the first report measuring lifetime values of protein condensates. The observed bi-exponential decay is consistent with prior studies on free tryptophan in solution, which attribute the two components (∼0.5 ns and ∼3.1 ns) to the existence of rotational isomers, one where the protonated amino group (NH₃⁺) is in close proximity to the indole ring, enhancing intramolecular quenching and shortening the excited-state lifetime, and another where the NH₃⁺ is further away, resulting in reduced quenching and a longer lifetime^61^. This model was further validated using N-acetyl-L-tryptophanamide (NATA), a neutral analog that exhibits a single exponential decay with a lifetime of 3.1 ns^61^. However, tryptophan fluorescence is also susceptible to quenching by nearby side chains, such as those of glutamine, asparagine, acidic residues (protonated Asp/Glu), cysteine, and histidine, complicating direct structural interpretation^62^. Reported lifetimes in single-Trp proteins vary considerably depending on their local structure, from 7.2/2.9 ns in phospholipase A2 to 3.6/1.1 ns in glucagon^63,64^. In our data, the short-component lifetime in p53C comparing with p63 and p73 monomers, support the idea of greater local flexibility and solvent exposure of its tryptophan residue, in contrast to the more buried environments observed in p63C and p73C.

The NMR data reveal distinct mechanistic responses to perturbation within the p53 family core domains. Among the paralogs, p53C exhibits the highest conformational sensitivity to hydrostatic pressure, manifested by widespread chemical shift perturbations, non-reversible signal attenuation, and pressure-induced aggregation. This behavior contrasts sharply with p63C, which undergoes spatially localized and fully reversible unfolding transitions, and with p73C, which largely preserves global structural integrity under comparable conditions. Direct comparison of hydrostatic pressure and chemical denaturation further highlights these differences. While pressure induces diffuse, heterogeneous destabilization and irreversible aggregation in p53C, consistent with non-cooperative and hydration-driven unfolding involving multiple partially unfolded intermediates, guanidine hydrochloride produces a more localized response centered on the β-sandwich core and Zn²⁺-binding region, without signatures of nonlinear or heterogeneous behavior. These observations demonstrate that hydrostatic pressure uniquely exposes the non-cooperative and spatially heterogeneous unfolding landscape of p53C, whereas chemical denaturation primarily probes global thermodynamic stability. Such differences reflect underlying disparities in core stability and dynamic regulation across the p53 family, with the enhanced thermal stability of p63C likely arising from a more effective hydrophobic gating that shields both faces of the β-sandwich from solvent intrusion.

Although p53C and p73C display comparable thermal stability and partially overlapping frustration profiles (Figs. 1K, S3 and S14), their responses to hydrostatic pressure reveal clear mechanistic differences. While both paralogs are less thermally stable than p63C (Fig. 1K), p73C exhibits a markedly higher resistance to hydrostatic pressure, maintaining reversible chemical shift perturbations, minimal signal attenuation, and overall structural integrity across the pressure range examined (Figs. 3F and 4E, F). In contrast, p53C undergoes heterogeneous, non-cooperative pressure-induced destabilization, characterized by widespread signal loss at higher pressures and irreversible aggregation upon decompression (Fig. 4A, B). Together, these results indicate that the aggregation propensity of p53C does not arise from globally reduced stability per se, but rather from pressure-sensitive, hydration-driven unfolding pathways that are largely suppressed in p73C. The dI/dP analysis further supports this divergence by revealing that p53C exhibits a heterogeneous and segmental response to pressure, in contrast to the more uniform profiles of p63C and p73C. This uneven pressure sensitivity reflects localized differences in conformational plasticity and hydration susceptibility across the p53C sequence. Notably, many residues with high dI/dP values coincide with solvation patches and frustration-enriched loops, suggesting that these regions act as mechanically labile nodes that promote conformational destabilization. Such local unfolding may expose hydrophobic or amyloidogenic segments, priming the protein for aggregation. The broader and skewed dI/dP distribution of p53C is therefore not only indicative of structural fragility but also mechanistically linked to its heightened propensity to form amyloid-like aggregates under stress. In contrast, the more compact and symmetric dI/dP patterns in p63C and p73C likely reflect evolved protection against such transitions, underscoring the selective pressure to minimize aggregation in these paralogs.

Nonlinear pressure-dependent ¹H chemical shift behavior has been established as a sensitive marker of sparsely populated excited conformational states that become thermodynamically accessible under compression, particularly in regions associated with cavities or enhanced hydration^52,53,65–67^. These states correspond to locally expanded and more solvent-penetrated conformations rather than globally unfolded species. In p53C, residues exhibiting nonlinear pressure responses are located near solvation-sensitive surface patches, suggesting that pressure promotes access to locally perturbed conformations with altered packing and hydration. While such excited states are transient and low in population, their repeated sampling may increase the probability of exposing aggregation-prone surfaces under destabilizing conditions. Thus, nonlinear chemical shift behavior provides a residue-specific readout of local conformational heterogeneity that may be relevant for understanding early aggregation events.

In our analysis, we identified nonlinear behavior in selected residues of p53C, notably S99, L130, T155, and D184. These residues also exhibit elevated solvent-accessible surface area and volume compared to their homologs in p63C and p73C, indicating increased topographical exposure. These dynamic features are less pronounced in the paralogs, supporting the notion that p53C maintains a conformationally labile ensemble that enhances both functional adaptability and aggregation susceptibility.

Backbone ^15^N relaxation measurements further support this view. At 150 bar, a marked decrease in average R₂ and in the R₂/R₁ ratio was observed for p53C, indicating increased fast-timescale (ps–ns) motion and a global loss of tertiary contacts. Residue-specific shifts in R₂/R₁ values also revealed a redistribution of slower-timescale (μs–ms) exchange processes, suggesting a pressure-induced remodeling of the conformational energy landscape. These transitions may expose otherwise shielded segments of the core, notably the amyloidogenic ILTIITL motif^29,68^, which showed line broadening even under mild pressure. Despite its low solvent accessibility under native conditions, the behavior of I251 under pressure indicates that this buried sequence may act as a latent aggregation nucleus upon destabilization.

It is important to clarify that patches P1–P6 do not correspond to amyloidogenic sequences themselves, but rather define regions of altered protein–water interactions and local structural heterogeneity that modulate the stability of the p53C fold. Per-residue MDDF analysis reveals that these patches exhibit distinct hydration patterns relative to p63C and p73C (Fig. 2E–M). Among these regions, patch P3 (S3–S4 loop) stands out as a convergence point of altered solvation, elevated energetic frustration, and low sequence conservation uniquely in p53C (Fig. S3B–E), consistent with its enhanced local conformational plasticity.

Importantly, the use of intra-protein Z-score normalization reveals that regions displaying elevated frustration in p53C are statistically enriched relative to its own energetic background and map to poorly conserved, highly solvated surface patches, whereas the corresponding homologous regions in p63C and p73C occupy neutral or minimally frustrated regimes (Fig. S14). This quantitative framework supports the interpretation that evolutionary divergence in p53 selectively tolerated higher local frustration in exchange for functional plasticity.

Notably, residues within or adjacent to P3 and P5 exhibit pronounced pressure sensitivity in NMR measurements, including diffuse signal attenuation (Fig. 4A, B), asymmetric dI/dP distributions (Fig. 4F), and nonlinear ¹H chemical shift responses (e.g., L145, T155, and D184; Figs. 5A, B and S15), indicating transient local destabilization under compression. In contrast, the canonical amyloidogenic ILTIITL segment (residues 251–257) remains largely shielded, displaying mostly linear pressure-dependent chemical shifts (Fig. 5A, B), although early line broadening at I251 under mild pressure suggests that aggregation-prone motifs may become dynamically accessible only after hydration-driven perturbations occur elsewhere in the fold.

Together, these observations support a hierarchical model in which poorly conserved, hydration-sensitive surface patches act as gateways for pressure-induced water infiltration and local unfolding, thereby facilitating the eventual exposure of latent aggregation nuclei rather than serving as direct aggregation hotspots themselves. Structural and functional analyses have emphasized that p53 aggregation is driven not by a single amyloidogenic segment but rather by multiple, spatially distributed nucleation sites across the core domain. Wang and colleagues demonstrated that several regions, particularly those near DNA-binding loops and β-strands, can independently contribute to aggregation, supporting a modular model of amyloid assembly in p53C^69^. In our study, we observed that local conformational plasticity, transient solvation exposure, and high energetic frustration in solvation patch 3 coincide with the previously described p53 multisite aggregation mechanism^69^, further suggesting that dynamic accessibility is a key determinant to nucleate aggregation (Fig. 6). This reinforces the idea that interventions aimed at stabilizing local structural environments, reducing solvation-induced flexibility, or shielding energetically frustrated residues may provide more comprehensive aggregation control than single-site targeting approaches.

The mechanistic framework identified here is directly relevant to well-characterized cancer-associated p53 mutations. Hotspot variants such as Y220C, R175H, R248Q, and M237I destabilize the p53 core domain through convergent mechanisms involving altered hydration, energetic frustration, and enhanced access to transient states. The mutation Y220C, for example, creates transiently-populated states with a highly unstable cavity^70,71^. Fluorescence and thermodynamic analyses demonstrate that R175H populates solvent-exposed, aggregation-prone conformations, consistent with a rugged conformational landscape. DNA-contact mutations such as R248Q, positioned in loop L3, disrupt local hydrogen-bonding networks and reduce thermodynamic stability, producing localized structural and dynamical perturbations without complete global unfolding^6^. These effects are consistent with the population of alternative conformational substates, as discussed in integrative folding analyses of p53 mutants^72^. Similarly, the glioblastoma-associated M237I mutation has been reported to populate destabilized and more hydrated conformational states, exhibit increased hydrophobic exposure, and form pre-amyloidogenic and amyloid-like species, indicating an enhanced susceptibility to solvent-mediated destabilization^6,26^. Notably, many of these mutations map to regions that, in wild-type p53C, already exhibit elevated solvation and energetic frustration, suggesting that oncogenic mutations may amplify pre-existing dynamic vulnerabilities.

In protein evolution more generally, enhanced functional adaptability is often associated with stability–evolvability trade-offs, where sequence changes that expand functional landscapes can come at the cost of reduced thermodynamic/kinetic robustness^73^. Within the p53 family, this trade-off is plausibly expressed through paralog-specific sequence divergence that reshapes local hydration and energetic landscapes in surface-exposed, weakly conserved segments: in p53, the same “tunable” regions that may facilitate conformational sampling needed for stress-responsive tumor suppression could also increase sensitivity to hydration-driven destabilization and misfolding, whereas p63/p73, whose ancestral/expanded roles emphasize developmental and germline programs, may be under stronger selection for structural resilience in the core fold^74,75^.

Together, these findings highlight a dynamic and structurally heterogeneous core in p53C that contrasts with the more rigid frameworks of p63C and p73C (Fig. 6). The capacity of p53C to populate transiently disordered or excited states in which solvation takes place may enable a broader range of biological interactions but also predisposes it to misfolding and aggregation. This dynamic plasticity, modulated by surface hydration and a hydrophobic gating, provides a structural basis for the divergent behaviors of p53 family members under stress and may help explain the unique aggregation propensity of p53 in cancer-associated conditions.

## Supplementary information

Transparent Peer Review file

Supplementary Information

Description of Additional Supplementary Files

Reporting summary

MOESM5_ESM

## Data Availability

All data supporting the findings of this study are available within the article and its Supplementary Information. Molecular dynamics input files, initial and final coordinates, representative trajectories, and custom scripts used for hydration, frustration, and pressure-response analyses are available from the corresponding authors upon reasonable request. The source data are provided in Supplementary Data 1.
