# Supplementary material for: Cancer-linked aggregation of p53 is driven by sequence-encoded frustration, solvation, and hydrophobic gating absent in its paralogs
Source: Commun Chem. 2026 May 16;9:266. doi: 10.1038/s42004-026-02050-2 (PMC13438141; doi:10.1038/s42004-026-02050-2)
Supplement: Supplementary file 1 [file 42004_2026_2050_MOESM1_ESM.pdf]

nature portfolio

# Peer Review File

# Cancer-linked aggregation of p53 is driven by sequence-encoded frustration, solvation, and hydrophobic gating absent in its paralogs

Corresponding Author: Professor Guilherme de Oliveira

**This file contains all reviewer reports in order by version, followed by all author rebuttals in order by version.**

Version 0:

Reviewer comments:

Reviewer #1

(Remarks to the Author)
General Comment

The manuscript entitled“Sequence-encoded solvation of frustrated residues and altered hydrophobic gating drive aggregation disparities in the p53 family” by Andrade et al. presents a detailed residue-level analysis of the biophysical and dynamic structural properties of p53 and its family proteins, integrating macroscopic spectroscopic measurements—such as fluorescence, fluorescence lifetime, and light scattering—with advanced molecular dynamics simulations, hydration analysis, frustration analysis, and high-pressure NMR spectroscopy. In particular, the authors employ state-of-the-art NMR techniques, including relaxation measurements under high pressure, to achieve a comprehensive elucidation of these properties. The authors' rigorous and methodical approach to this challenging subject is highly commendable.

However, the descriptions of these rigorous data are highly narrative but without numeric evidence and appropriate statistical analysis. Thus, this reviewer finds it difficult to regard this work as a fully completed scientific paper; rather, it gives the impression of an unfinished draft. The following are the major issued for further improvement in both the explanations based on some detailed data and statistics, and the representations of cross-protein comparisons.

1. Limited Quantitative and Normalized Comparison of Hydration Analysis

The manuscript provides descriptive and graphical representations of hydration patterns across p53 family members; however, quantitative or normalized comparisons are not included. Systematic metrics (such as Z-scores or per-residue standardized hydration levels) and statistical evaluations would be necessary to enable direct, objective cross-protein comparisons. As it stands, the discussion of differential hydration remains qualitative, and the conclusions are not sufficiently supported by reproducible, quantitative evidence.

2. Limited Normalization and Quantitative Comparison in Frustration Analysis

Although the standard frustration index is cited, the analysis is presented primarily in qualitative or narrative form. The manuscript would benefit from including tabulated or statistically summarized frustration Z-scores or other normalized parameters across homologous positions of p53, p63, and p73. Without such data, it is difficult for readers to independently assess the extent or significance of the proposed differences in local frustration among these proteins. Probably this issue is most difficult, since the original authors of the frustration analysis did not recommend comparing frustration indices across different proteins. The authors may newly develop a sophisticated normalization method to achieve this point.

3. Qualitative Treatment of HPP-NMR Cavity Analysis Without Normalized Comparison

The high-pressure NMR (HPP-NMR) results are similarly described in qualitative terms, with visual annotations rather than normalized or statistically aggregated metrics. Incorporating consistent quantitative comparisons? such as normalized dI/dP values, systematic summaries of chemical shift perturbations, or statistical analyses across homologous residues?would greatly strengthen the interpretation of differential cavity responses among the p53 family members.

4. Potential Over-interpretation of p53 and p73 Stability and Aggregation Propensity

The conclusion that p53 is uniquely aggregation-prone, and thereby distinct from p73, is not convincingly supported by the presented data. Both p53 and p73 appear to share comparable thermal stability, HPP-NMR responses, and frustration profiles, whereas p63 emerges as the distinctly more stable paralog. The manuscript would benefit from a more balanced discussion that acknowledges this similarity, as the current interpretation risks overstating the uniqueness of p53's biophysical behavior and pathological implications.

Overall comment:

Taken together, these issues reduce the quantitative rigor, transparency, and logical coherence of the manuscript's central conclusions. Addressing them through additional quantitative analyses and more systematic cross-protein comparisons would substantially enhance the clarity and scientific robustness of the work.

Reviewer #2

(Remarks to the Author)
The manuscript titled "Sequence-encoded solvation of frustrated residues and altered hydrophobic gating drive aggregation disparities in the p53 family" by Andrade et al. addresses an important, longstanding question as to the unique ability of p53 to undergo misfolding and aggregation compared to its paralogs, namely p63 and p73. The authors have used a diverse array of tools involving time-resolved fluorescence spectroscopy, high-pressure NMR, and molecular dynamics simulations to make a compelling case for presenting the inherent structural and dynamic features of p53, as opposed to the other paralogs. These studies demonstrate that the structurally heterogeneous population of p53 contains transient disordered states and exposed solvation patches that drive its misfolding and aggregation observed under disease conditions. This is a great piece of work that is of interest to this journal, and I recommend its publication. The authors might want to address my minor comments as summarized below.
The authors state the fluorescence lifetimes of Trp in the second decimal place (pages 19, 34, and Figure 1), which I believe is not correct up to the second decimal place. They can round it up to the first decimal place and state the typical error. The changes are very small for both tau1 (0.7-0.9 ns) and tau2 (3.1-3.4 ns). Such changes could also be ascribed to the addition of 15% polyethylene glycol (due to the changes in the viscosity and refractive index). Also, it would be good to state the time-response/resolution of their frequency-domain lifetime measurements in the Methods section on page 10. I believe frequency-domain measurements have a slower response compared to TCSPC measurements.

Reviewer #3

(Remarks to the Author)
This manuscript presents a comprehensive comparison of the DNA-binding domains of p53, p63, and p73 to elucidate why p53—but not its paralogs—is uniquely prone to pathological misfolding and aggregation. Using an impressive combination of fluorescence spectroscopy, high-pressure and chemical-denaturation NMR, molecular dynamics, and local energetic frustration analysis, the authors identify sequence-encoded solvation patterns, loop-specific hydration anomalies, and differences in hydrophobic gating that define p53's aggregation susceptibility. They further show that these structural and dynamic distinctions are evolutionarily encoded and correlate with non-conserved, highly frustrated surface patches that destabilize p53 under pressure or partial denaturation.
Overall, the work offers strong mechanistic insight into the molecular origins of p53's conformational fragility and provides a valuable framework for understanding aggregation-linked tumorigenesis and rational design of stabilizing interventions.
Major Strengths
1. Important and timely biological problem
The disproportionate aggregation propensity of p53 relative to p63/p73 is fundamental to understanding cancer-related loss-of-function and dominant-negative mechanisms. The manuscript provides new and mechanistically coherent explanations for this phenomenon.

2. Integrated experimental-computational strategy
The study combines light scattering, fluorescence lifetime, pressure NMR, GdnHCl NMR, molecular dynamics simulations, and frustration analysis. This multidimensional approach allows the authors to correlate hydration, flexibility, energetic frustration, and unfolding transitions with exceptional clarity.
3. Discovery of solvation patches and hydration-driven instability
The identification of six non-conserved solvation patches (P1–P6) and their correlation with pressure-enhanced hydration, dynamic heterogeneity, and early unfolding is a notable and novel contribution.
4. Mechanistic link between local frustration and aggregation
The detailed frustration maps reveal that p53 harbors a cluster of highly frustrated contacts particularly in patch 3, consistent with its weaker structural integrity and aggregation propensity.
This connection between solvation, frustration, and hydrophobic gating strengthens the paper's central thesis.
5. Strong comparative framework
The parallel assessment of p53C, p63C, and p73C—using identical perturbations—provides a compelling narrative of evolutionarily encoded structural divergence that balances flexibility against stability.
6. High relevance for therapeutic and diagnostic development
The work provides insights that could guide design of next-generation p53 stabilizers, small molecules, and aggregation

inhibitors, and may support early diagnostic strategies based on detection of p53 aggregates.
Major Points for Improvement
1. Expand the discussion of why evolutionary divergence produced these solvation patterns
The manuscript clearly demonstrates distinct hydration and frustration features but could better articulate the evolutionary trade-offs—e.g., functional flexibility vs. structural stability—and their consequences for tumor suppression.

2025Nov_Review report_p53
2. More explicit integration of solvation patches with specific aggregation hotspots
The paper describes patches P1–P6 in detail, but the connection between each patch and experimentally observed aggregation-prone regions, including the ILTIITL segment, could be more explicitly drawn.
3. Clarify the interpretation of pressure-versus-chemical denaturation
A table or schematic comparing the onset of signal loss, CSP distributions, and unfolding behavior for each protein under pressure vs. GdnHCl would help readers appreciate the unusual non-cooperative, heterogeneous unfolding of p53C.
4. Strengthen discussion of cavity formation and nonlinear CSP behavior
The sections referencing Akasaka-type nonlinear shifts are compelling but could be expanded to clarify how these sparsely populated excited states relate to aggregation nucleation.
Minor Revisions
1. Author list punctuation and affiliation formatting should be reviewed for consistency.
2. The symbol “*” indicating corresponding authors is missing in the affiliation list.
3. Introduce p53C / p63C / p73C at first use to avoid confusing non-specialist readers.
4. Standardize terminology: avoid alternating between “p63/73C” and “p63C and p73C.”
5. Figure 1R: include scale bars for DIC images.
6. In several figure legends, clarify whether fluorescence changes correspond to unfolding, aggregation, or both.
7. Consider briefly commenting on the implications for known cancer-associated mutants (e.g., Y220C, R248Q, R175H) in the Discussion, as this will increase translational impact.
Recommendation
I recommend the manuscript for publication after minor revision.
This is a rigorous, well-executed, and conceptually strong study that provides high-resolution mechanistic insight into the differential aggregation propensities of the p53 family. The combination of NMR, simulation, and frustration analysis is particularly powerful, and the identification of solvation-encoded instability patches is scientifically valuable. With minor clarifications and expanded discussion, this work will have significant impact in structural biology, biophysics, and cancer research.

Reviewer #4

(Remarks to the Author)
I co-reviewed this manuscript with one of the reviewers who provided the listed reports. This is part of the Communications Chemistry initiative to facilitate training in peer review and to provide appropriate recognition for Early Career Researchers who co-review manuscripts.

Version 1:

Reviewer comments:

Reviewer #2

(Remarks to the Author)
The revised manuscript can now be accepted for publication.

Reviewer #3

(Remarks to the Author)
The authors have adequately addressed the comments of all reviewers. The paper can now be published.

Reviewer #4

(Remarks to the Author)
I co-reviewed this manuscript with one of the reviewers who provided the listed reports. This is part of the Communications Chemistry initiative to facilitate training in peer review and to provide appropriate recognition for Early Career Researchers who co-review manuscripts.

**Open Access** This Peer Review File is licensed under a Creative Commons Attribution 4.0 International License, which permits use, sharing, adaptation, distribution and reproduction in any medium or format, as long as you give appropriate credit to the original author(s) and the source, provide a link to the Creative Commons license, and indicate if changes were made.
In cases where reviewers are anonymous, credit should be given to 'Anonymous Referee' and the source.
The images or other third party material in this Peer Review File are included in the article's Creative Commons license, unless indicated otherwise in a credit line to the material. If material is not included in the article's Creative Commons license and your intended use is not permitted by statutory regulation or exceeds the permitted use, you will need to obtain permission directly from the copyright holder.
To view a copy of this license, visit https://creativecommons.org/licenses/by/4.0/

Response to the Reviewers

Manuscript title (revised):
Cancer-linked aggregation of p53 is driven by sequence-encoded frustration, solvation, and hydrophobic gating absent in its paralogs

Former title:
Sequence-encoded solvation of frustrated residues and altered hydrophobic gating drive aggregation disparities in the p53 family

Manuscript ID: COMMSCHEM-25-0946-T

Dear Editor and Reviewers,

We sincerely thank the Editor and the Reviewers for their thorough, constructive, and insightful evaluation of our manuscript. We believe that the comments and suggestions have substantially strengthened the rigor, clarity, and impact of this work.

Before addressing the specific comments raised by the reviewers, we would like to note that, in response to the reviewers' feedback and to improve clarity and precision, we have revised the title of the manuscript. The new title emphasizes the cancer-linked nature of p53 aggregation, clarifies the mechanistic drivers identified in this study, and explicitly highlights the absence of these features in its paralogs. We believe this revised title more accurately reflects the central conclusions of the work and aligns better with the scope and readership of Communications Chemistry.

In response to the reviewers' comments, we have performed a major revision of the manuscript that includes the addition of quantitative and normalized analyses for hydration, local frustration, and HHP-NMR data, the inclusion of new supplementary figures and tables enabling direct cross-paralog comparisons and expansion of the Methods section.

**Reviewer #1**

General comment

We thank Reviewer #1 for the careful and detailed assessment of our work. We agree that the original version relied too heavily on descriptive interpretations in some sections and that additional quantitative analyses were necessary to support cross-protein comparisons. All concerns have been fully addressed, as detailed below.

1. Limited quantitative and normalized comparison of hydration analysis

Response:
We fully agree with this assessment. To address this limitation, we performed an additional quantitative analysis of hydration using the existing molecular dynamics data. Per-residue water densities were calculated and normalized using Z-scores for each protein, allowing direct comparison across homologous positions in p53C, p63C, and p73C, independent of global sequence differences or solvent box size. These results are now presented in Supplementary Figure S13 and Supplementary Table 2, and the Results section has been revised accordingly.

2. Limited normalization and quantitative comparison in frustration analysis

Response:
We agree that direct comparison of raw frustration indices across different proteins is conceptually problematic. To address this concern, we implemented an intra-protein normalization strategy based on Z-scores, which preserves the relative energetic landscape within each protein while avoiding cross-protein bias. Quantitative summaries of normalized frustration values, contact counts, and the fraction of highly frustrated interactions are now provided in Supplementary Table 3 and Figure S14, enabling objective and reproducible assessment of differential frustration patterns.

3. Qualitative treatment of HPP-NMR cavity analysis

Response:
We extracted quantitative metrics from the HHP-NMR datasets by calculating pressure coefficients for chemical shift variations using linear and quadratic regression across the applied pressure range. These results are summarized in Supplementary Table 4 and Figure S15, and the Results section was revised to incorporate these quantitative comparisons.

4. Potential over-interpretation of p53 stability and aggregation propensity

Response:
We thank the reviewer for this important point and agree that p63C emerges as the most thermostable paralog. We have revised the text to clarify that the distinction between p53C and p73C does not arise from gross differences in thermal stability or overall frustration levels, which indeed show partial overlap between these two proteins. Instead, our conclusions are based on the mechanistic nature of their responses to perturbation.

While p53C and p73C exhibit comparable thermal aggregation onset temperatures and broadly similar frustration distributions, their behavior under hydrostatic pressure differs markedly. High-pressure NMR data reveal that p53C undergoes heterogeneous, non-cooperative signal loss across the domain and forms irreversible aggregates upon decompression, whereas p73C largely retains spectral reversibility and structural integrity

under comparable pressure conditions. This distinction is further supported by the distribution of pressure-sensitive residues and nonlinear chemical shift responses, which in p53C preferentially localize within or near solvation-sensitive surface patches.

Accordingly, we have revised the manuscript to emphasize that the aggregation propensity of p53C reflects not a uniquely low global stability relative to p73C, but rather the presence of hydration-sensitive, mechanically labile regions that promote non-cooperative unfolding pathways under pressure. This revised framing acknowledges the biophysical similarities between p53C and p73C, while highlighting the specific mechanistic features that distinguish p53C and underpin its heightened susceptibility to irreversible aggregation.

**Reviewer #2**

We thank Reviewer #2 for the positive evaluation and helpful technical suggestions.

Fluorescence lifetimes

Response:
We agree that reporting lifetimes to the second decimal place overstates the precision of frequency-domain measurements. All lifetime values were therefore rounded to one decimal place, and the instrumental temporal resolution (~0.1 ns) is now explicitly stated in the Methods section.

**Reviewer #3**

We thank Reviewer #3 for the very positive and insightful evaluation of the manuscript.

Expanded evolutionary discussion

Response:
The Discussion section was expanded to address evolutionary trade-offs between functional flexibility and structural stability across the p53 family.

Integration of solvation patches and aggregation hotspots

Response:
We explicitly linked solvation patches, particularly patch P3, to known aggregation-prone segments such as the ILTIITL motif.

Pressure versus chemical denaturation

Response:
A new comparative summary is provided in Supplementary Table 1, highlighting differences in structural response under hydrostatic pressure and chemical denaturation.

Nonlinear chemical shifts

Response:
The discussion of nonlinear chemical shift behavior was expanded to clarify its relationship to low-population excited states and early aggregation events.

Minor revisions

Response:
All requested editorial and figure revisions were implemented.

Molecular dynamics checklist and editorial requirements

The molecular dynamics simulation checklist has been fully completed and submitted. The Methods section has been expanded accordingly, and Data Availability and Code Availability sections have been added.

Final remarks

We believe that these revisions fully address the reviewers' concerns and significantly strengthen the manuscript. We thank the Editor and Reviewers for the opportunity to improve this work and hope that the revised version is now suitable for publication in Communications Chemistry.

Sincerely,

Guilherme Caldas de Andrade
