# Supplementary material for: Cancer-linked aggregation of p53 is driven by sequence-encoded frustration, solvation, and hydrophobic gating absent in its paralogs
Source: Commun Chem. 2026 May 16;9:266. doi: 10.1038/s42004-026-02050-2 (PMC13438141; doi:10.1038/s42004-026-02050-2)
Supplement: Supplementary file 2 [file 42004_2026_2050_MOESM2_ESM.pdf]

Cancer-linked aggregation of p53 is driven by sequence-encoded frustration, solvation, and hydrophobic gating absent in its paralogs

Guilherme C. de Andrade[1,2], Ander F. Pereira[3], Paulo H. B. Ferreira[4], Karen D. da Cruz[1,2], Gileno S. de Sousa[1,2], Michelle F. Mota[1,2], Ronaldo J. de Oliveira[4], Leandro Martínez[3], Mayra A. Marques[1,5], Jerson L. Silva[1,6*], Guilherme A. P. de Oliveira[1,2*]

[1] Institute of Medical Biochemistry Leopoldo de Meis, National Institute of Science and Technology for Structural Biology and Bioimaging, National Center of Nuclear Magnetic Resonance Jiri Jonas, Federal University of Rio de Janeiro, Rio de Janeiro, RJ. 21941-902, Brazil.

[2] Laboratory of Structural Bioengineering Jerson Silva, Institute of Medical Biochemistry Leopoldo de Meis, Federal University of Rio de Janeiro (UFRJ), Rio de Janeiro, RJ, 21941-902, Brazil.

[3] Institute of Chemistry and Center for Computing in Engineering & Science, State University of Campinas (UNICAMP), 13083-861, Campinas, SP, Brazil.

[4] Laboratório de Biofísica Teórica, Department of Physics, Institute of Exact, Natural Sciences and Education, Federal University of the Triângulo Mineiro, Uberaba, MG, Brazil.

[5] Faculty of Pharmacy, National Institute of Science and Technology for Structural Biology, Federal University of Rio de Janeiro (UFRJ), RJ, 21941-902, Brazil.

[6] D'Or Institute for Research and Education (IDOR), Rio de Janeiro, Brazil, 22281-100.

*Corresponding author:
Guilherme A. P. de Oliveira, gaugusto@bioqmed.ufrj.br
Jerson L. Silva, jerson@bioqmed.ufrj.br

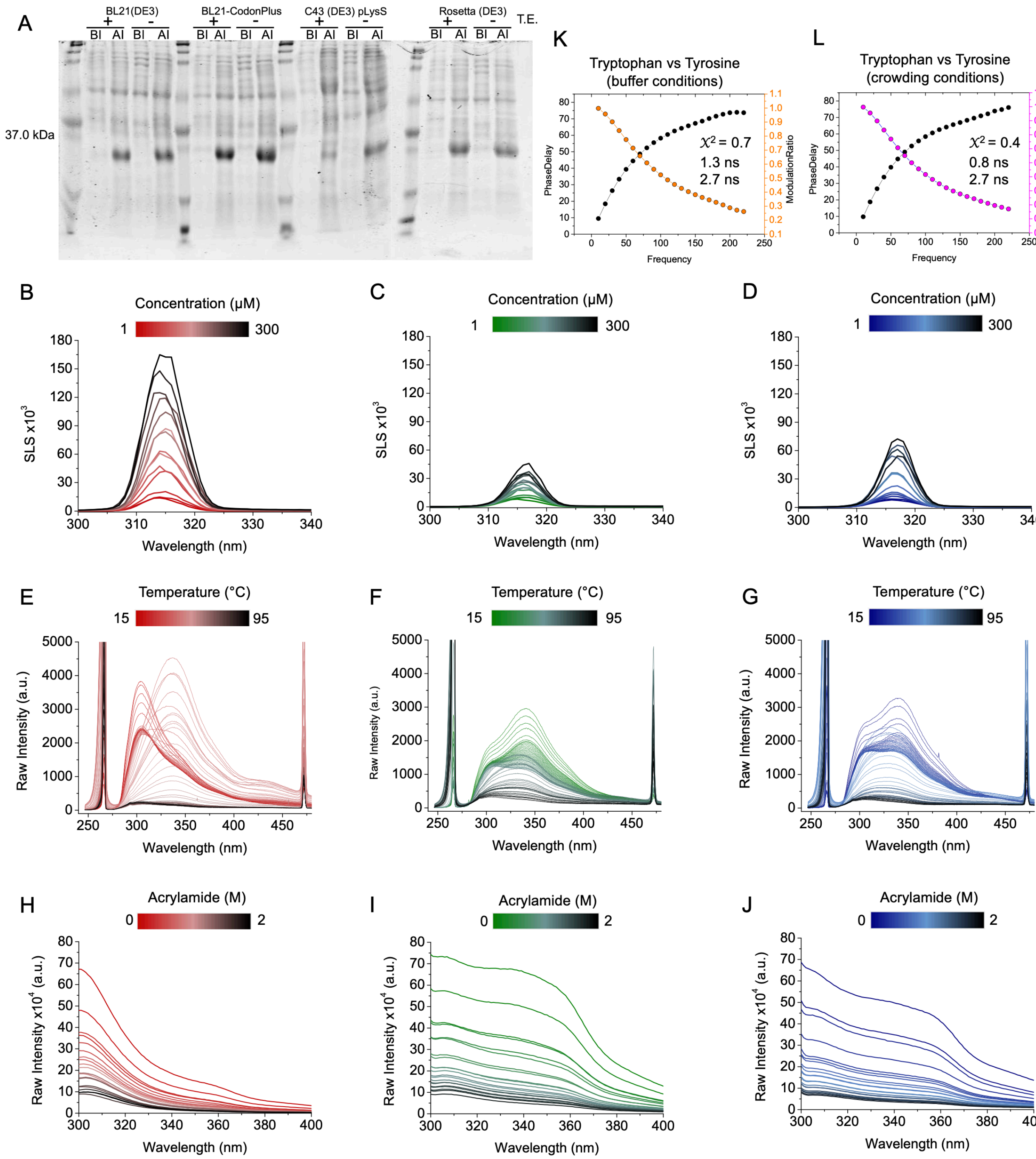

Figure S1. Optimization of protein yields and raw fluorescence data.
(A) SDS-PAGE gel stained with Coomassie blue showing p53C expression levels in four different *E. coli* strains, before (BI) and after (AI) IPTG induction, in the absence (–) or presence (+) of trace elements (T.E.). (B–D) Line plots of static light scattering profiles as a function of increasing protein concentration. (E–G) Raw fluorescence emission spectra acquired using the UNCLe platform across a temperature gradient. (H–J) Raw fluorescence spectra obtained using an ISS spectrofluorometer in the presence of increasing acrylamide concentrations. Color coding: red for p53C, green for p63C, and blue for p73C. (K, L) Dual Y-axis plots showing control experiments for fluorescence lifetime measurements.

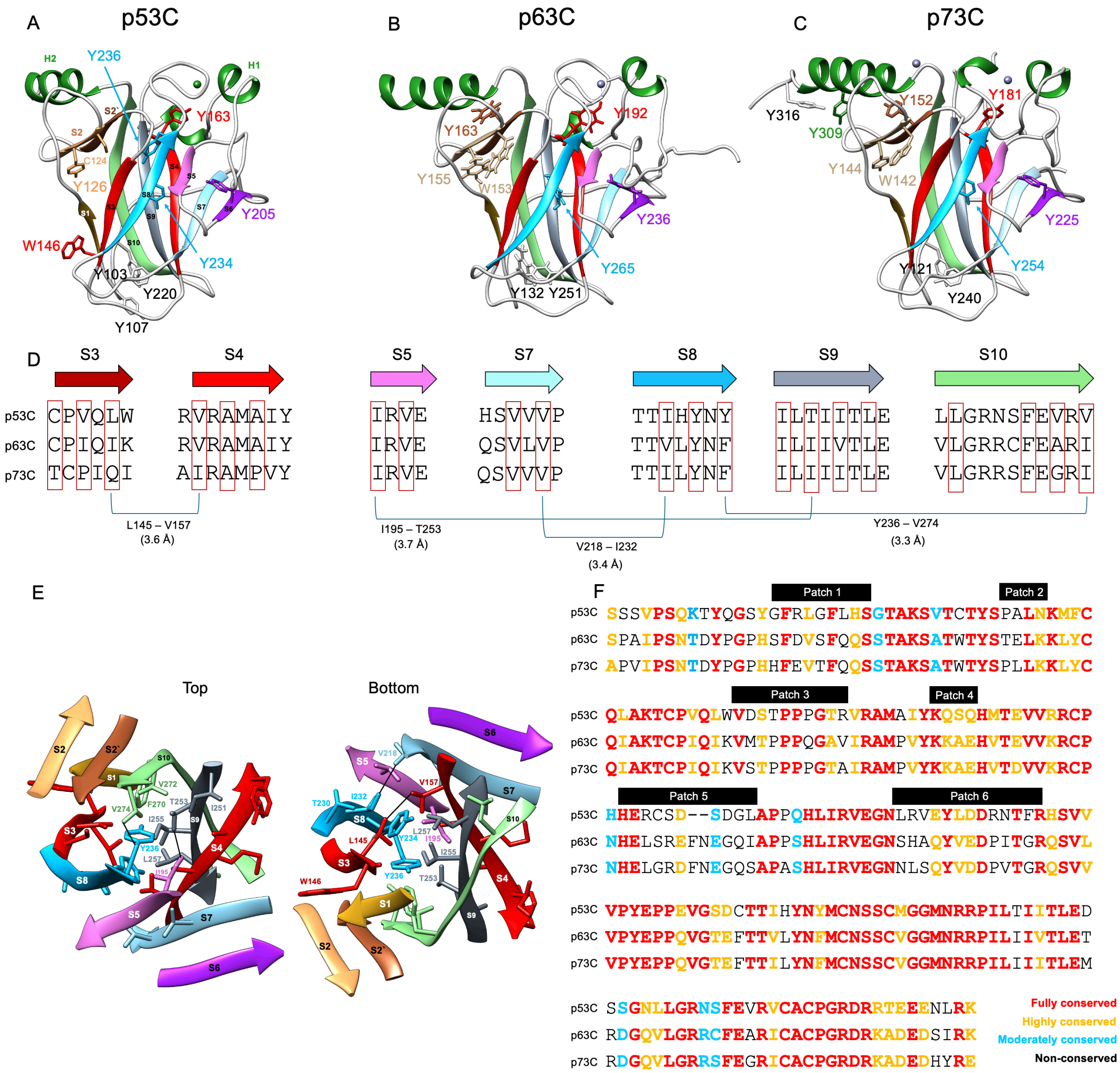

Figure S2. Structural analysis.

Ribbon representations of (A) p53C, (B) p63C, and (C) p73C highlighting tyrosine (Y) distribution and the single tryptophan (W), shown as sticks. Secondary structure elements are color-coded, and loops are shown in gray.

(D) Residues involved in β-strands within the p53 family. Red rectangles indicate residues facing the inner hydrophobic β-sandwich core. Horizontal brackets represent short-range contacts.

(E) Ribbon representation of the p53C inner hydrophobic core with hydrophobic residues shown as sticks. Hydrophobic contacts are indicated by black lines. Secondary structure elements (S) denote strands; loops were omitted for clarity.

(F) Sequence alignment of p53C, p63C, and p73C showing conservation across the p53 family and the location of solvation patches.

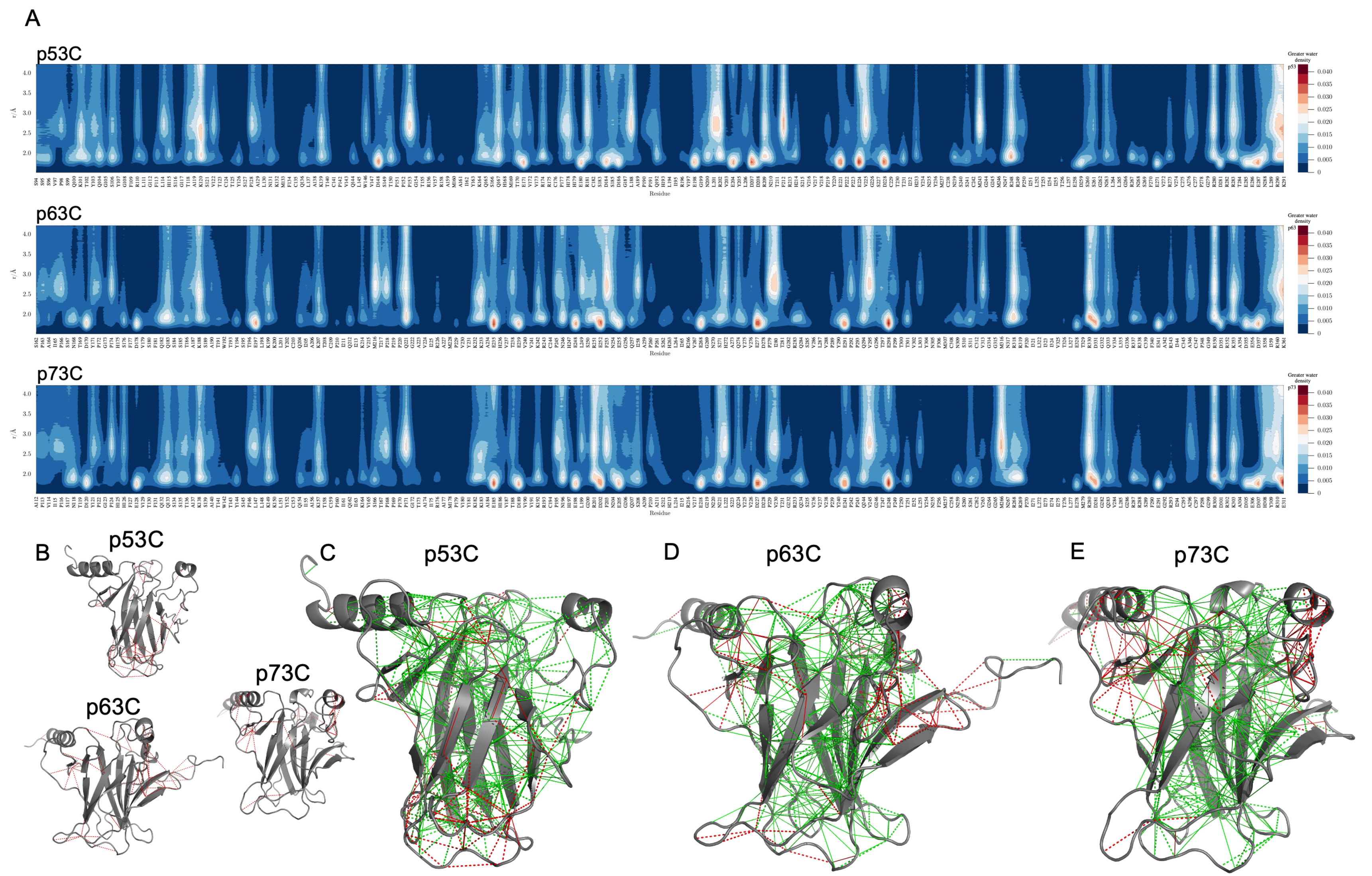

Figure S3. Hydration and frustration analysis. (A) Color maps showing the spatial density of water molecules mapped onto p53C, p63C, and p73C residues. (B-E) Ribbon representations of p53C, p63C, and p73C showing minimally frustrated (green) and highly frustrated (red) contact pairs. Panel B shows only highly frustrated contact pairs for clarity.

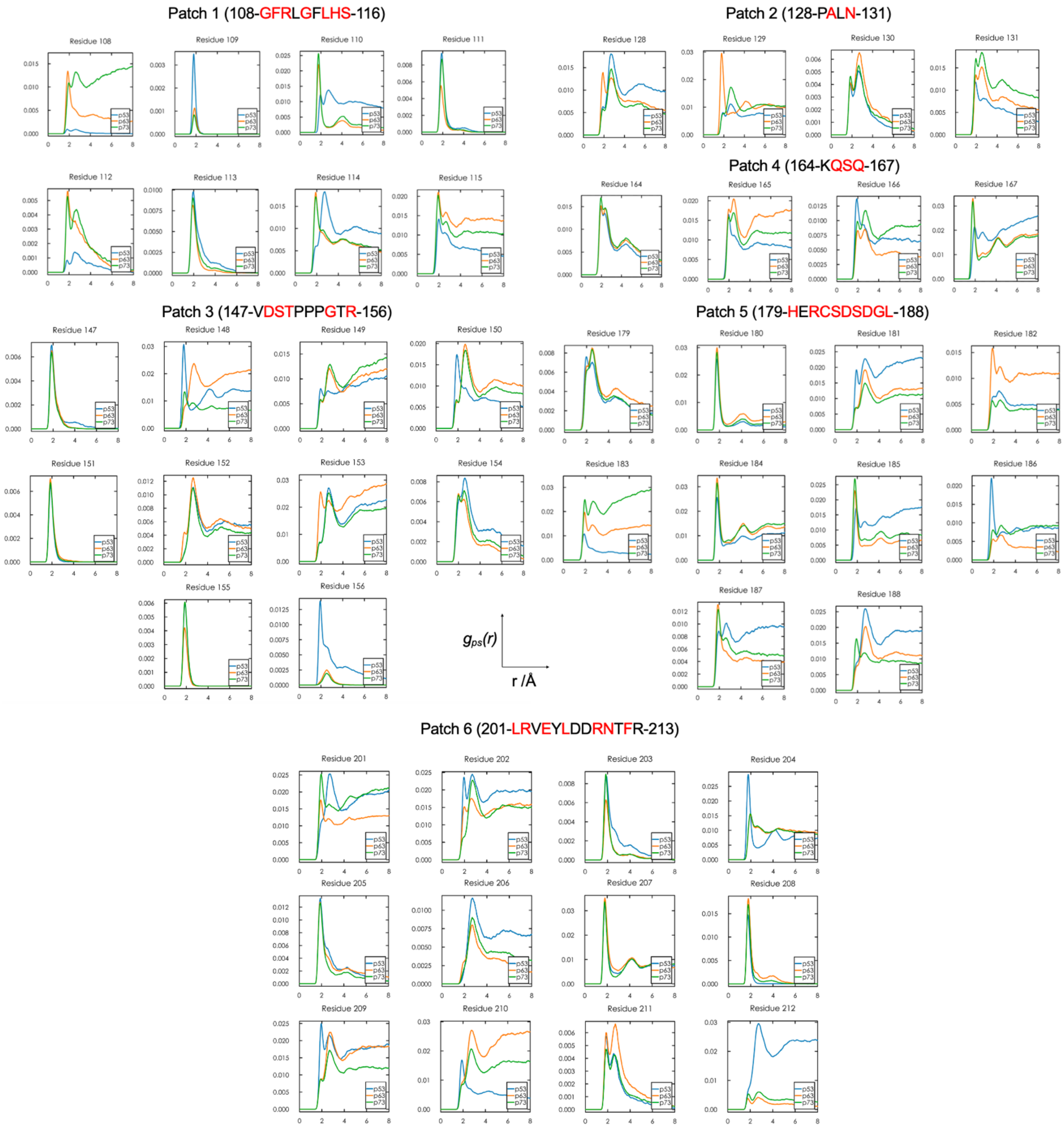

Figure S4. Minimum-distance distribution function per residue. Collection of line plots show the contribution of each residue from patches P1 to P6 in p53C, p63C, and p73C to the water MDDF.

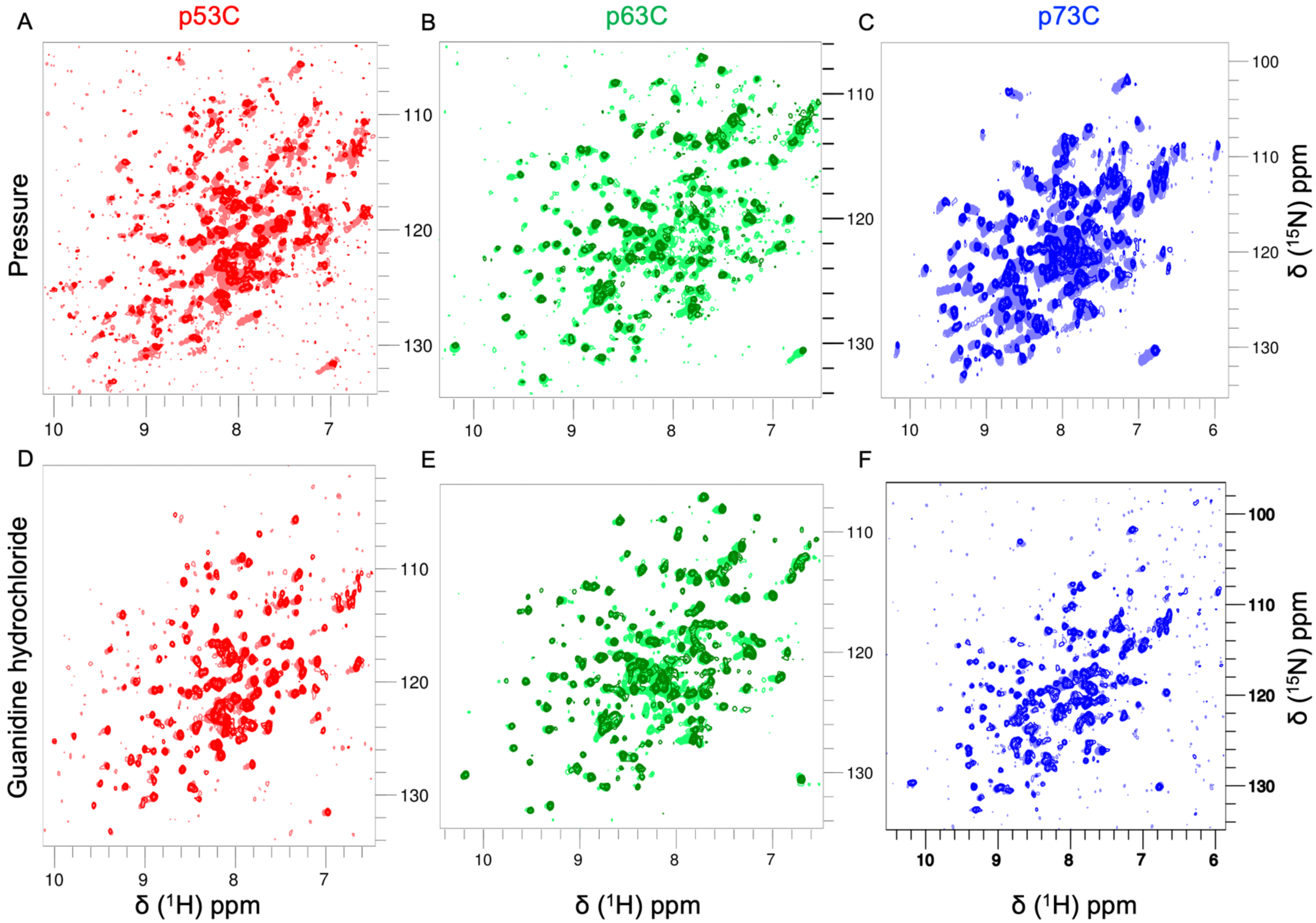

Figure S5. Pressure and guanidine titrations monitored by nuclear magnetic resonance.
Overlaid two-dimensional ^{1}H–^{15}N TROSY-HSQC spectra acquired during titrations at increasing (A-C) hydrostatic pressures and (D -F) guanidine hydrochloride concentrations. Initial spectra are shown in red, green, and blue for p53C, p63C, and p73C, respectively. Incremental titration points are displayed in progressively lighter shades of the corresponding colors.

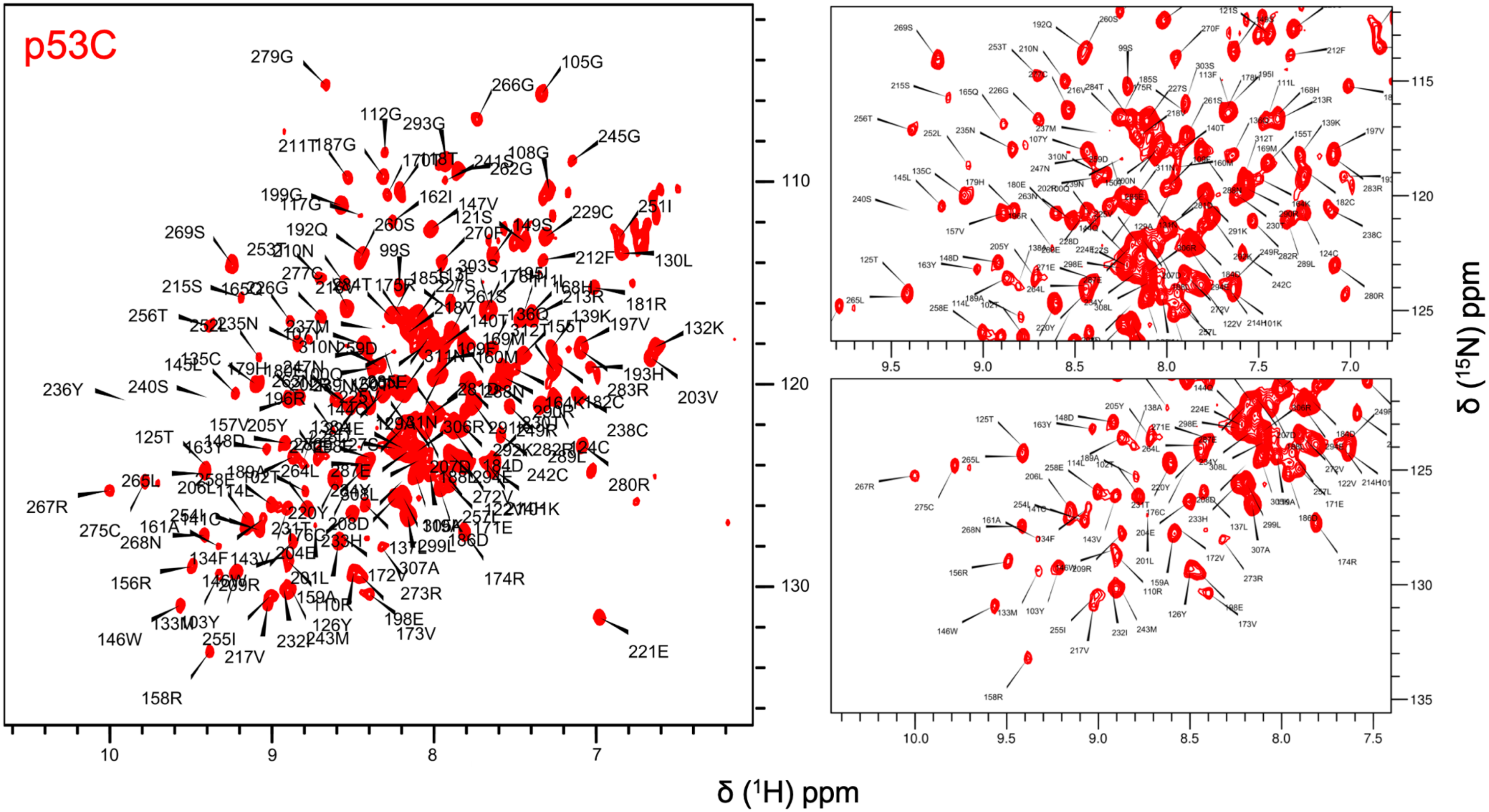

Figure S6. Assigned ^{1}H-^{15}N TROSY-HSQC spectra of p53C at 25°C.

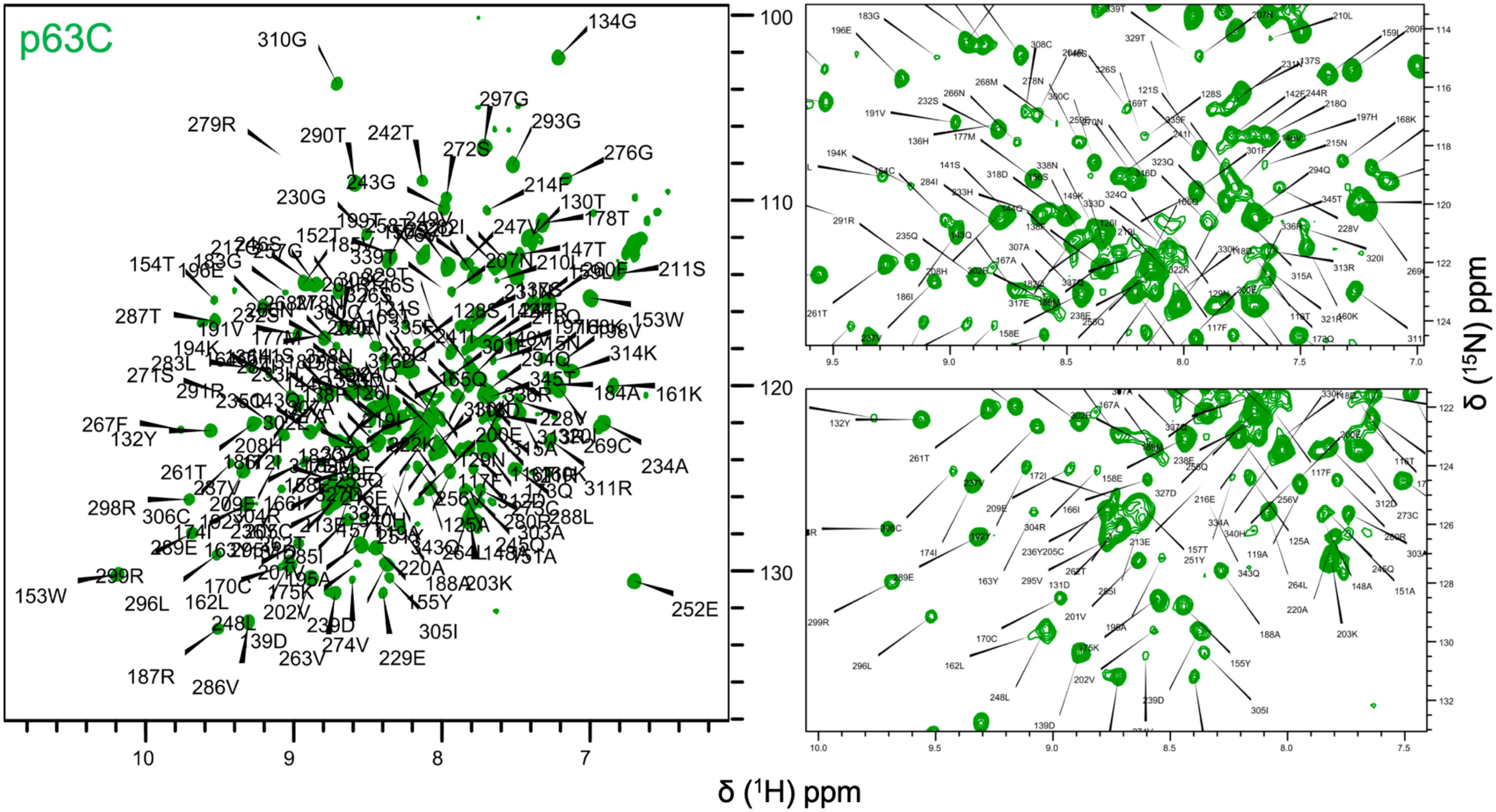

Figure S7. Assigned ^{1}H-^{15}N TROSY-HSQC spectra of p63C at 25°C.

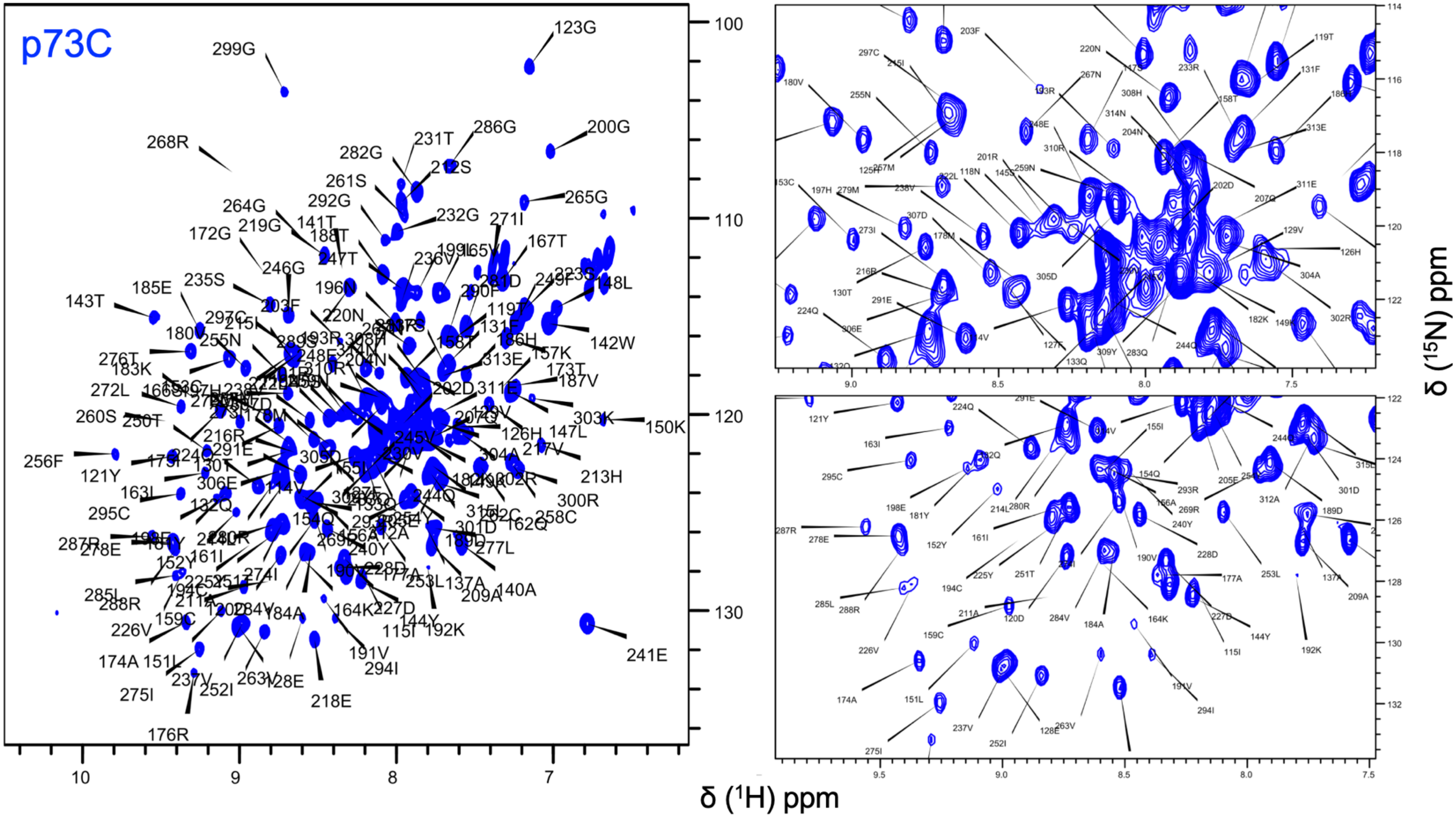

Figure S8. Assigned ^{1}H-^{15}N TROSY-HSQC spectra of p73C at 25°C.

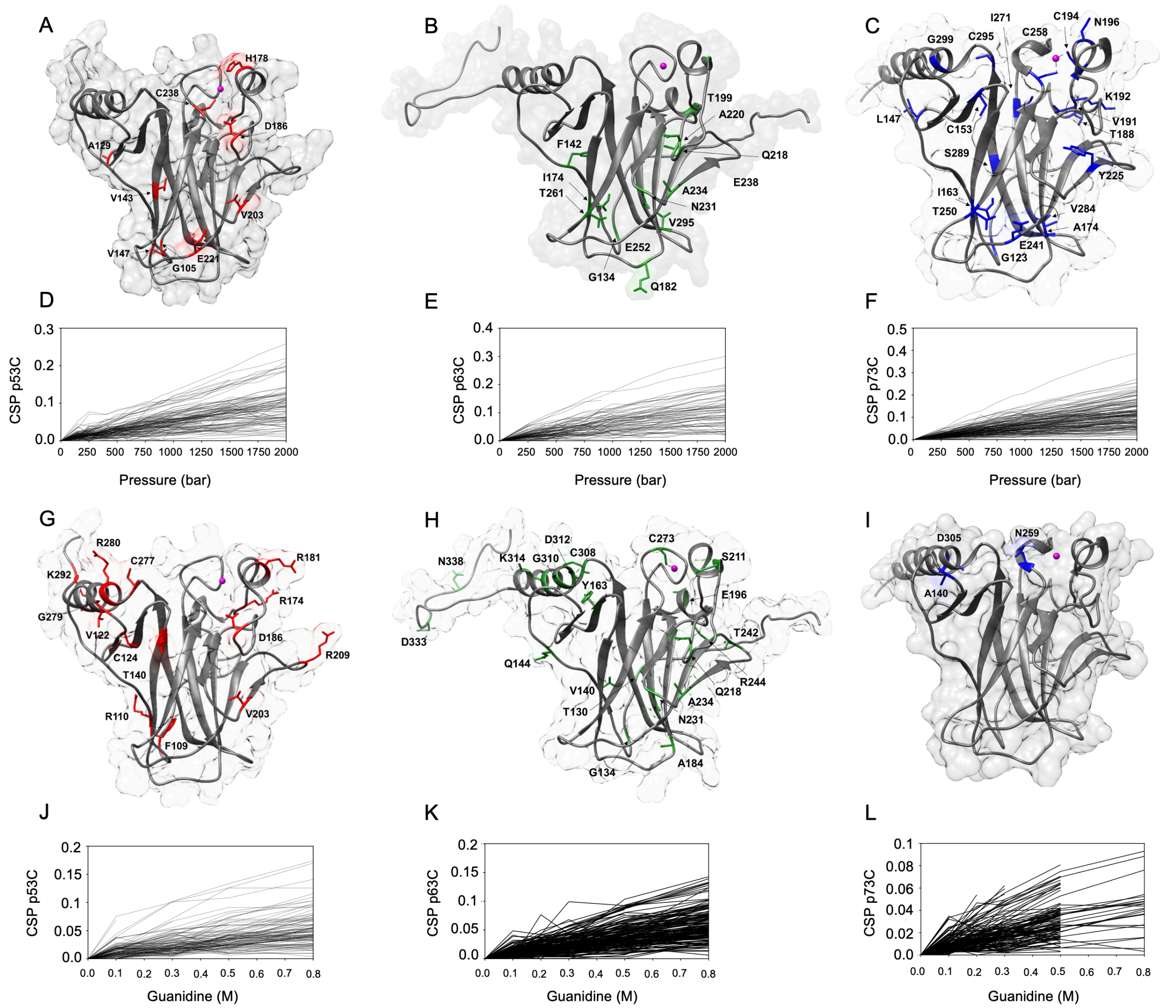

Figure S9. Chemical shift analysis.
Ribbon and surface representations highlighting residues with significant chemical shift perturbations ($\Delta\delta > 1\sigma$) following (A–C) pressure titration and (G–I) guanidine titration, shown as colored sticks. Chemical shift perturbation (CSP) plots for all assigned residues are shown for (D–F) pressure titration and (J–L) guanidine titration, relative to spectra acquired at 1 bar and 0 M denaturant, respectively. The color code is red for p53C, green for p63C, and blue for p73C. Zinc ion is shown as a magenta sphere.

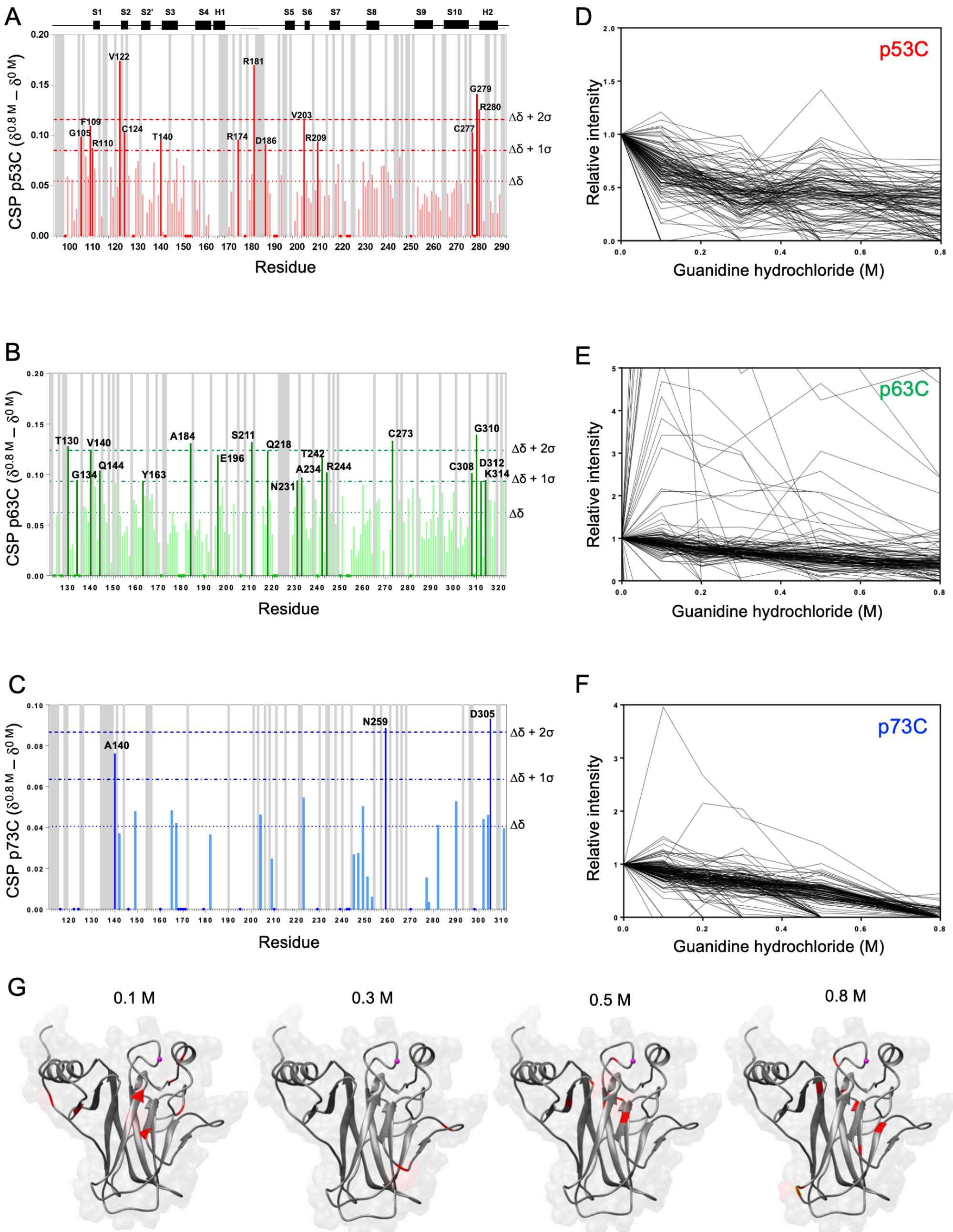

Figure S10. Chemical shift perturbation (CSP) and intensity profile during guanidine titration. (A-C) Bar plots showing CSP values for 0.8 M vs. 0 M guanidine hydrochloride, plotted as a function of residue number for (A) p53C, (B) p63C, and (C) p73C. Dotted, dash-dotted, and dashed lines represent the average (Δδ), average + 1 standard deviation (Δδ + 1σ), and average + 2 standard deviations (Δδ + 2σ), respectively. Ambiguous or unassigned H–N correlations are shown in gray; proline residues are depicted as circles. Secondary structure elements are indicated by black rectangles at the top. (D-F) Line plots showing the relative signal intensities of each assigned residue, normalized to the spectrum acquired in the absence of denaturant. (G) Ribbon and surface representation of p53C highlighting in red the residues whose signals disappeared at the corresponding guanidine concentration.

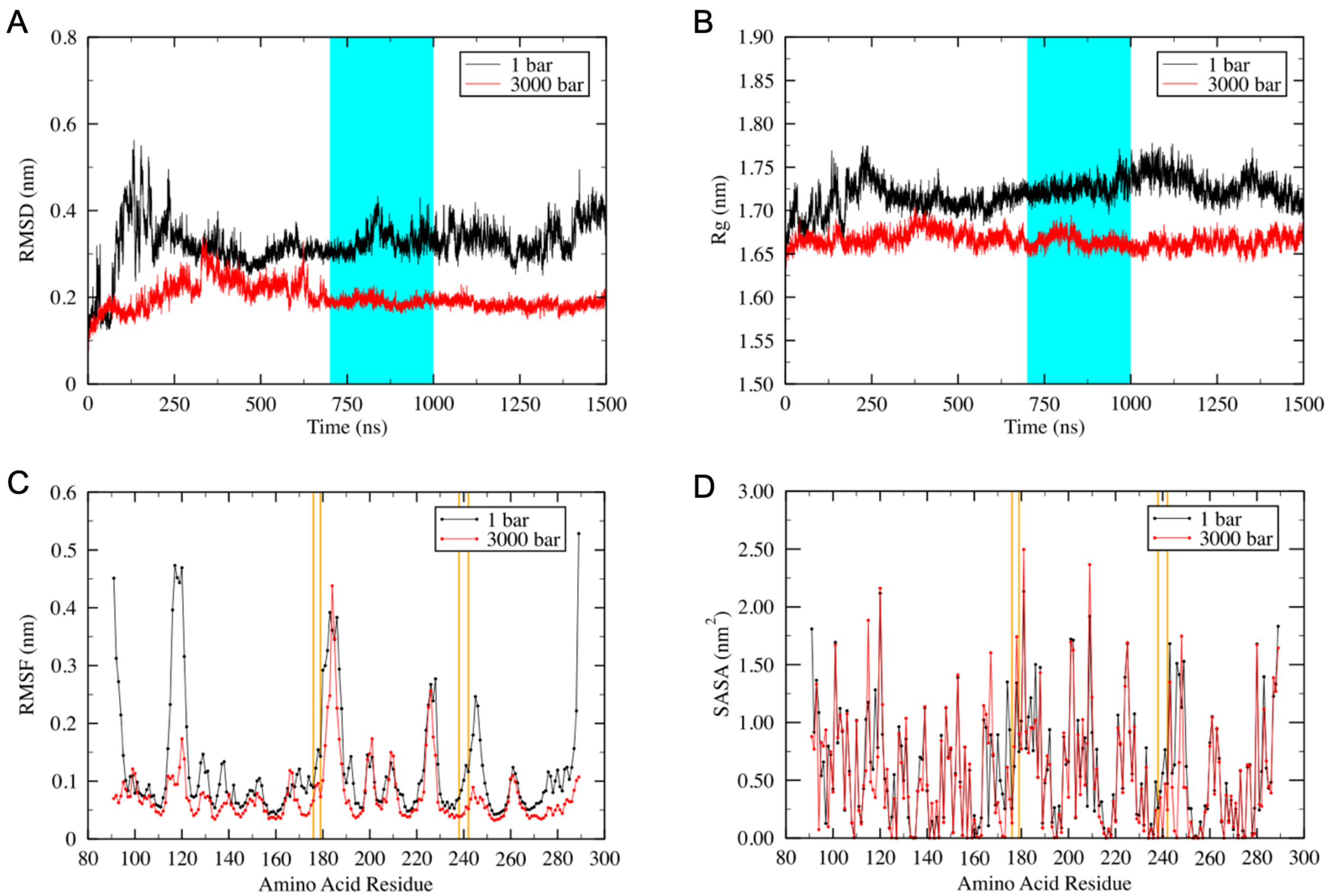

Figure S11. Results from molecular dynamics (MD) simulations of p53C. (A) Backbone root mean square deviation (RMSD) for p53 residues 91–289 over the course of the simulation.

(B) Radius of gyration (Rg) calculated for p53 residues 91–289.

(C) Root mean square fluctuation (RMSF) of Cα atoms for residues 91–289.

(D) Average solvent-accessible surface area (SASA) per residue for the same segment and time window. The time interval highlighted in cyan (700–1000 ns) in panels (A) and (B) indicates the region used to compute RMSF and SASA in panels (C) and (D), respectively. Residues involved in Zn^{2+} coordination are highlighted in orange in panels (C) and (D).

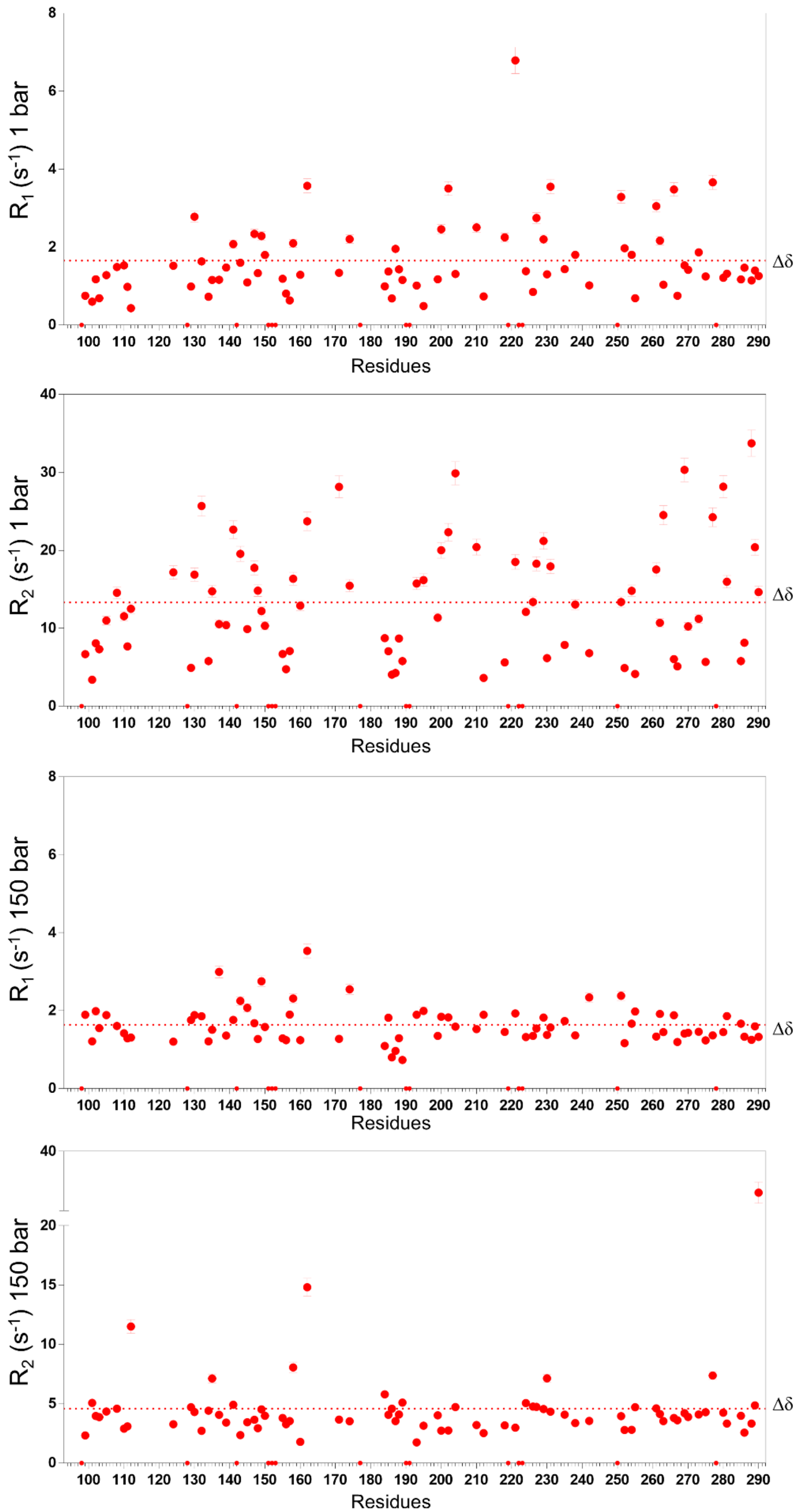

R1 (s-1) 1 bar
Residues
Δδ
R2 (s-1) 1 bar
Residues
Δδ
R1 (s-1) 150 bar
Residues
Δδ
R2 (s-1) 150 bar
Residues
Δδ

Figure S12. ^{15}N relaxation data of p53C at 1 and 150 bar.
Dot plots showing the longitudinal (R_1) and transverse (R_2) relaxation rates as a function of residue number for p53C at 1 and 150 bar. Red dotted lines indicate the average values. Error bars represent measurement uncertainties (see Methods).

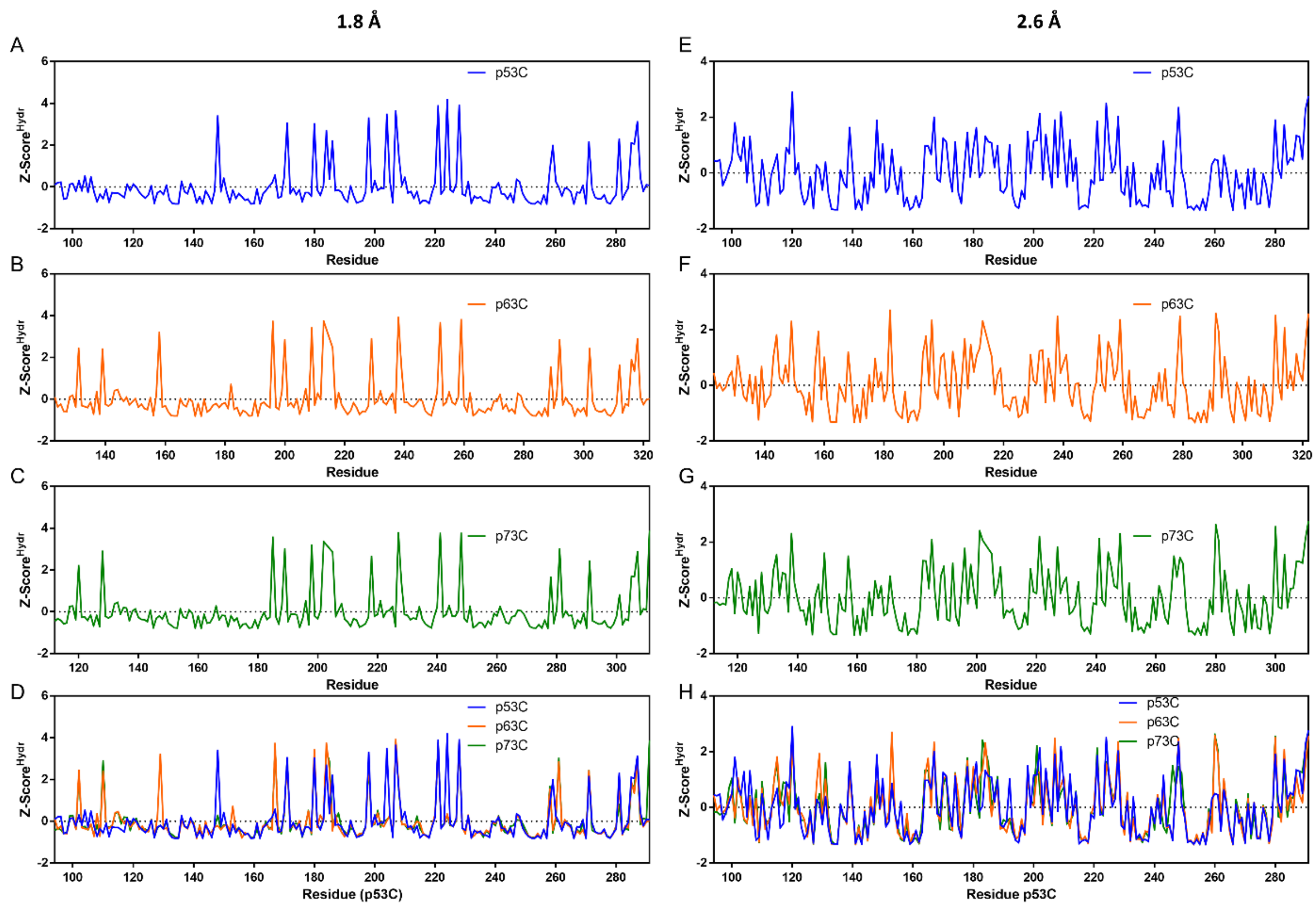

Figure S13. Normalized per-residue hydration profiles of the p53 family core domains.
Residue-level hydration Z-scores (Z_score_Hydr) calculated from MDDF integrations for the first (1.8 Å; panels A–D) and second (2.6 Å; panels E–H) solvation shells. Panels A–C and E–G show individual profiles for p53C (blue), p63C (orange), and p73C (green), respectively, with Z-scores normalized within each protein. Panels D and H overlay the three paralogs mapped onto the p53C sequence index to facilitate direct comparison of homologous positions.

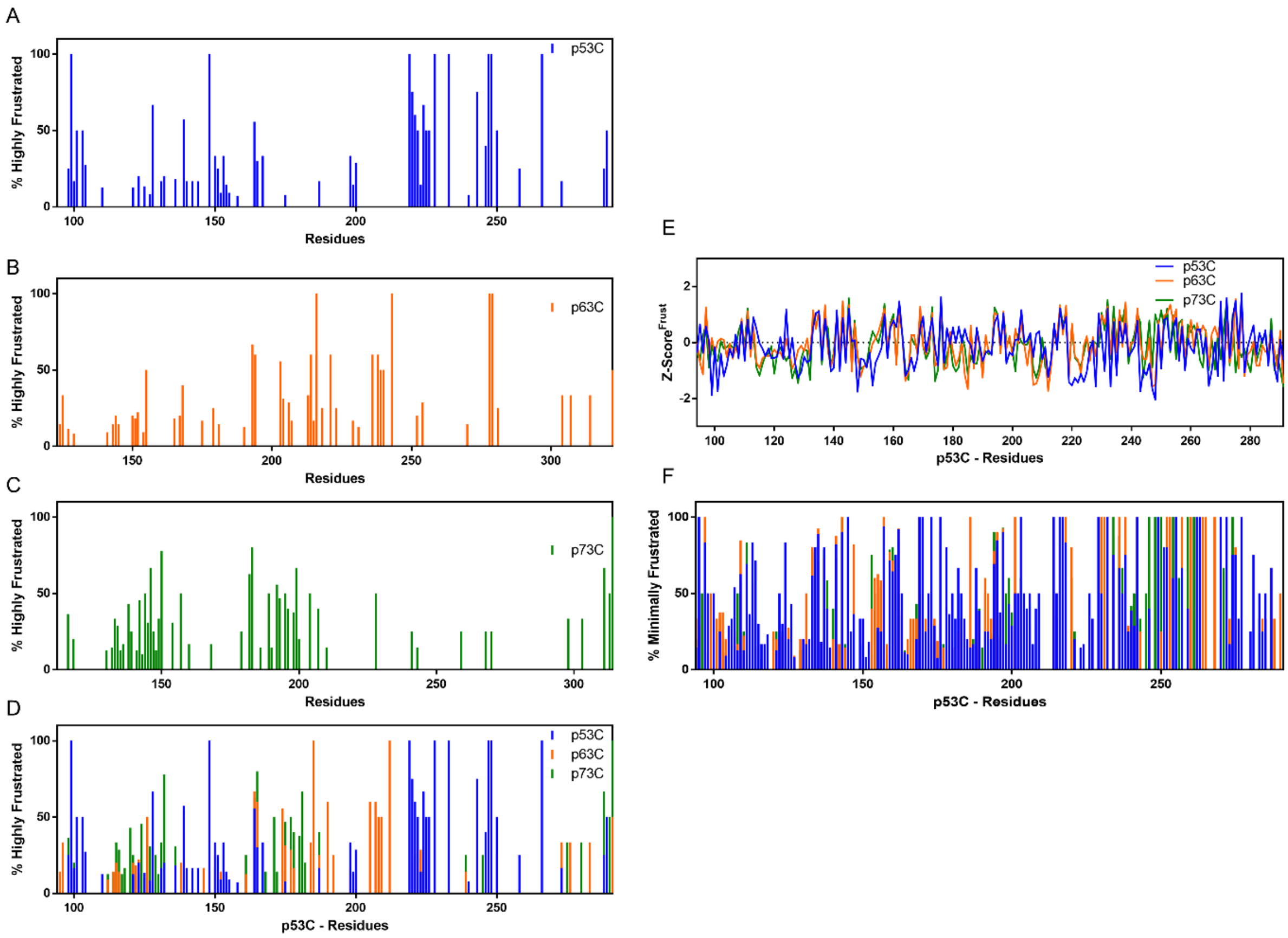

Figure S14. Residue-level energetic frustration profiles across the p53 family core domains. (A–C) Percentage of highly frustrated contacts per residue for p53C (A), p63C (B), and p73C (C), computed using the Frustratometer based on contact-based frustration indices. Bars represent the fraction of decoy contacts classified as highly frustrated at each residue position, highlighting localized regions of elevated energetic conflict within each protein. (D) Direct comparison of highly frustrated residues across p53C (blue), p63C (orange), and p73C (green), mapped onto the p53C sequence numbering to enable alignment of homologous positions. This representation reveals a higher density and clustering of highly frustrated residues in p53C relative to its paralogs. (E) Z-score–normalized frustration profiles for p53C, p63C, and p73C, obtained by internal normalization within each protein. (F) Percentage of minimally frustrated contacts per residue across the three proteins, illustrating the dominance of energetically optimized interactions in p63C and p73C compared to p53C.

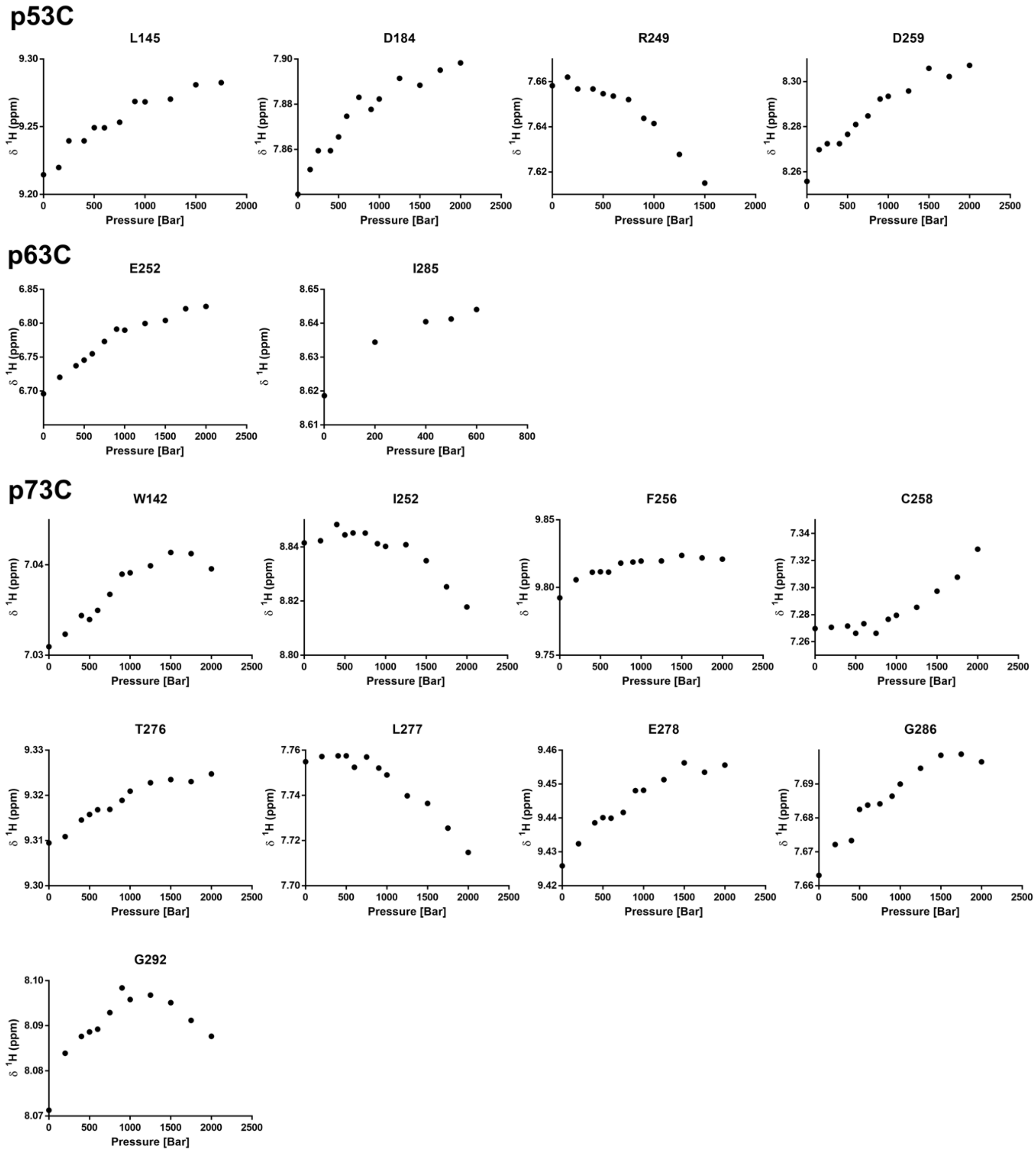

Figure S15. Nonlinear pressure-dependent ¹H chemical shift responses in selected residues of the p53 family core domains.

Representative pressure titration profiles of backbone amide ¹H chemical shifts (δ¹H) as a function of hydrostatic pressure are shown for residues in p53C (top row), p63C (middle row), and p73C (bottom rows). Pressure is reported in bar, and chemical shifts are given in ppm. These nonlinear responses are indicative of local structural rearrangements associated with hydration changes and internal cavity modulation.

Table S1. Comparative summary of hydrostatic pressure and chemical denaturation responses across the p53 family core domains.

	Hydrostatic pressure			Guanidine hydrochloride		
	**p53C**	**p63C**	**p73C**	**p53C**	**p63C**	**p73C**
**CSP distribution**	Heterogeneous, patch-localized	Broader but ordered shifts	Most extensive CSPs	Localized near β-sandwich core and Zn^{2+}-binding region	Distributed CSPs	Minimal CSPs (only three residues)
**Signal loss**	Broad range (≈1250–2000 bar), diffuse across domain	Early onset (≈250–750 bar), localized	Minimal; only three residues	Progressive with increasing GdnHCl concentration	High resistance, minor signal loss	Progressive signal loss at low concentrations (0.3–0.8 M)
**Reversibility**	Irreversible; aggregation observed after pressure release	Fully reversible	Fully reversible	x	x	x
**Unfolding behavior**	Non-cooperative, heterogeneous, multi-site destabilization	Ordered, stepwise unfolding	Largely resistant	Core-centered unfolding	Distributed	More chemically sensitive despite pressure resistance

Table S2. Simulation box details for each system from Cino et al (2016)[1].

Protein	Replicas	Box side (nm)	Water molecules	NaCl (mol L^{-1})	Total atoms
p53	2	7	10312	0.15	34084
p63	2	7	10313	0.15	34130
P73	2	7	10304	0.15	34094

Supplementary References

1 Cino, E. A., Soares, I. N., Pedrote, M. M., de Oliveira, G. A. & Silva, J. L. Aggregation tendencies in the p53 family are modulated by backbone hydrogen bonds. *Sci Rep* **6**, 32535, doi:10.1038/srep32535 (2016).
