# Supplementary material for: Cancer-linked aggregation of p53 is driven by sequence-encoded frustration, solvation, and hydrophobic gating absent in its paralogs
Source: Commun Chem. 2026 May 16;9:266. doi: 10.1038/s42004-026-02050-2 (PMC13438141; doi:10.1038/s42004-026-02050-2)
Supplement: Supplementary file 3 [file 42004_2026_2050_MOESM3_ESM.pdf]

Description of Additional Supplementary Files:

File name: Supplementary Data 1

Description: Source data underlying the main figures.
